# A Comparative Study of Deep Learning and Classical Modeling Approaches for Protein–Ligand Binding Pose and Affinity Prediction in Coronavirus Main Proteases

**DOI:** 10.1021/acs.jcim.5c02481

**Published:** 2025-12-22

**Authors:** Yue Liu, Haocheng Tang, Taoyu Niu, Junmei Wang

**Affiliations:** Department of Pharmaceutical Sciences and Computational Chemical Genomics Screening Center, School of Pharmacy, 6614 University of Pittsburgh, Pittsburgh, Pennsylvania 15261, United States

## Abstract

The accurate prediction of protein–ligand binding poses and affinities is central to structure-based drug design. In this study, we first benchmarked three distinct pose generation strategies for data sets from the ASAP Antiviral Challenge 2025: molecular docking (Glide and AutoDock Vina), ligand-based superposition (FlexS), and deep learning-based modeling (AlphaFold3, Boltz-2, DiffDock and Gnina). We evaluated their performance on binding pose prediction for ligands targeting SARS-CoV-2 and MERS-CoV main protease (Mpro). For binding affinity estimation, we implemented a machine learning-based scoring approach called ligand–residue interaction profile scoring function (LRIP-SF), which integrates molecular mechanics generalized Born surface area (MM-GBSA) energy decomposition with machine learning algorithms. Our results showed that deep learning-based modeling with AlphaFold3 achieved the highest pose prediction accuracy with a success rate of 88.1% and an average ligand root-mean-square deviation (LRMSD) of 1.12 Å. Moreover, binding poses predicted by AlphaFold3 enabled the most accurate potency predictions by LRIP-SF, with the lowest mean absolute error (MAE) and root-mean-square error (RMSE) in pIC_50_ units across both targets: the MAE and RMSE are 0.606 and 0.813, respectively, for MERS-CoV Mpro and 0.724 and 0.894 respectively for SARS-CoV-2 Mpro. Although ligand-based superposition method (FlexS) was less accurate in pose prediction, it offered competitive potency prediction performance with significantly lower computational cost. To interpret model predictions by LRIP-SF and identify critical binding determinants, we performed global sensitivity analysis (GSA), revealing key residues that contributed most significantly to ligand binding. These findings highlight the importance of pose quality and interaction profiling in affinity prediction and demonstrate the great potential of deep learning-based methods for drug discovery, especially in the absence of cocrystal structures.

## Graphical abstract

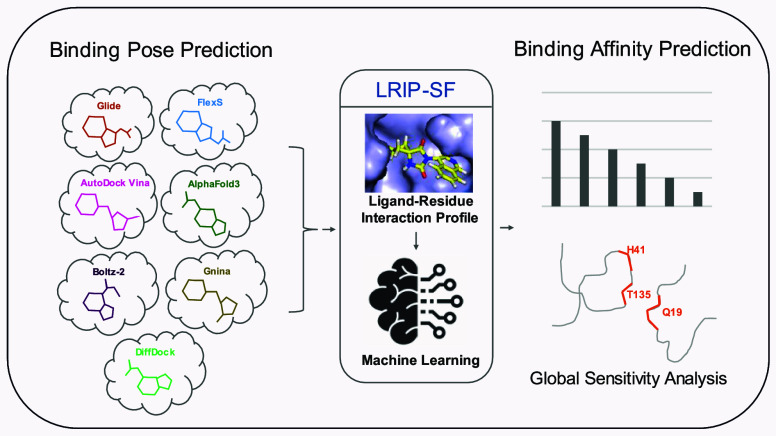

## 1 Introduction

The main protease (Mpro) of coronaviruses such as SARS-CoV-2 and MERS-CoV plays a critical role in viral replication by cleaving polyproteins into functional nonstructural proteins that form the replication–transcription complex.^1,2^ Owing to its essential function and the absence of closely related homologues in humans, Mpro has emerged as a highly attractive target for broad-spectrum antiviral drug development.^3^

To accelerate research in this area, the ASAP (AI-driven Structure-enabled Antiviral Platform) Discovery Consortium recently launched the Antiviral Competition, an open computational benchmarking challenge focused on SARS-CoV-2 and MERS-CoV Mpro.^4^ The challenge comprised three subchallenges: (1) ligand pose prediction,^5^ where participants predicted binding poses for compounds using SARS-CoV-2 Mpro crystal structures to guide modeling for both targets; (2) potency prediction,^6^ which involved predicting compound inhibitory potencies calculated using the fluorescence-based dose–response data; and (3) ADMET prediction,^7^ in which participants predicted absorption, distribution, metabolism, excretion, and toxicity end points based on public data sets.

We participated in both the pose and potency prediction subchallenges, initially employing a flexible ligand superposition method (FlexS) for our official submissions. After the challenge concluded, we refined our workflow to address pose alignment issues identified in the original results and expanded the analysis by incorporating additional computational methods. This postchallenge enhancement enabled a more comprehensive and comparative benchmarking of model performance across diverse approaches. In this posthoc evaluation, three distinct approaches were employed for ligand pose prediction. The first was molecular docking, a widely used technique that predicts ligand binding poses through scoring and sampling.^8^ We applied both Glide, a grid-based docking program with an empirical scoring function,^9^ and AutoDock Vina, which uses stochastic global optimization guided by a knowledge-based scoring function.^10^ The second approach was flexible ligand superposition, implemented using FlexS,^11,12^ which incrementally builds the conformation of a flexible test ligand based upon a rigid reference ligand by optimizing the spatial and physicochemical overlaps, including hydrogen bonding, charge, and hydrophobic features. This ligand-based method does not require protein structural data to exist and is especially suitable when only ligand data is available. The third approach is deep learning-based modeling, including AlphaFold3,^13^ Boltz-2,^14^ DiffDock^15^ and Gnina.^16^ Recently, deep learning-based methods have achieved significant progress in protein–ligand complex prediction. AlphaFold3 is a well-known deep learning framework capable of jointly modeling protein–ligand complexes directly from protein sequence and ligand SMILES representations, producing high-confidence poses without requiring prior crystal data.^13^ Boltz-2 is a generative diffusion model that formulates docking as a diffusion-based generative process, sampling ligand poses from a learned Boltzmann distribution conditioned on the receptor pocket.^14,17^ DiffDock uses a SE(3)-equivariant diffusion model to iteratively denoise ligand conformations in 3D space, achieving accurate and robust docking predictions.^15^ Gnina integrates convolutional neural networks into the AutoDock Vina framework for CNN-based pose scoring and refinement, combining deep learning with traditional docking principles.^16,18^

For ligand potency prediction, a variety of free energy simulation methods have been developed, such as pathway-based approaches represented by free energy perturbation (FEP) and thermodynamic integration (TI), which simulate gradual, nonphysical transformations between ligand states along an alchemical pathway using molecular dynamics or Monte Carlo sampling.^19,20^ These methods provide high accuracy but are computationally demanding. In contrast, end-point free energy methods, such as MM-GBSA, MM-PBSA, and linear interaction energy (LIE), estimate binding free energies from static snapshots of the bound and unbound states, offering a more computationally efficient alternative with reasonable predictive performance.^21–24^ These techniques are widely adopted in structure-based drug discovery for their practical balance between speed and accuracy.

To improve upon conventional approaches, we developed a machine learning-based scoring method called the ligand–residue interaction profile scoring function (LRIP-SF),^25^ which was applied by us in the potency prediction subchallenge. LRIP-SF leveraged detailed interaction profiles derived from MM-GBSA free energy decomposition, capturing the residue-level energetic contributions between the ligand and the protein. These interaction profiles served as input features for training machine learning models. To generate reliable predictions, the LRIP-SF workflow incorporated a structure minimization protocol with a generalized Born solvent model (MIN + GB) prior to LRIP calculation, resulting in accurate and computationally efficient binding affinity predictions. Because pose generation is a critical initial step in the LRIP-SF workflow, we adopted the same three strategies used in ligand pose prediction subchallenge (molecular docking with Glide and AutoDock Vina, flexible ligand superposition with FlexS, deep learning-based modeling with AlphaFold3, Boltz-2, DiffDock and Gnina) to generate ligand poses and systematically evaluated their respective performance in binding affinity prediction. We then applied a method called global sensitivity analysis (GSA)^26^ to interpret the best-performing model and identify key residues that contribute most significantly to ligand binding.

## 2 Methodologies

### 2.1 Pose Prediction

#### 2.1.1 Data Preparation

We used the data set provided by the ASAP Discovery Antiviral Ligand Pose Prediction Challenge 2025, hosted on the Polaris platform.^4^ Access to the data set required authentication via the Polaris CLI (polaris login), followed by programmatic access using the polaris Python API.^27^

The data set comprises 965 protein–ligand complexes, including 770 for SARS-CoV-2 Mpro in the training set, 98 for SARS-CoV-2 Mpro and 97 for MERS-CoV Mpro in the test set. Each data point contains a comprehensive set of molecular and structural information, as summarized in Table S1. Since the ligands in the test set were provided only in CXSMILES format, we first generated 3D conformations by converting each CXSMILES string into a MOL2 file using Open Babel v3.1.0.^28^ Then, all the input ligands were visually checked for chemical validity before modeling.

To support pose evaluation and structural alignment, the challenge also provided one reference complex structure for each drug target. These reference structures were used for aligning predicted poses and ensuring consistent coordinate frames across all submissions.^27^

#### 2.1.2 Molecular Docking with Glide

The ligands in the test set were docked to their corresponding receptors (as specified by the “protein label” column in Table S1) using the Glide docking program,^29^ implemented in the Schrödinger Suite (Maestro v11.2).^30^ Prior to docking, each protein target was prepared using the Protein Preparation Wizard in Maestro following standard protocols.^31^ This included removal of water molecules and cocrystallized solvents, addition of missing hydrogen atoms, etc. Geometry optimization was then performed using the OPLS force field,^32–34^ with only hydrogen atoms allowed to move. The catalytic dyad (His41–Cys145) was assigned automatically by Epik. These protonation forms were retained unchanged for all docking and scoring runs. Receptor grid files were then generated for each protein using the reference cocrystallized ligand provided by the challenge, to define the center of the binding pocket. No rotatable groups or constraint groups were defined during the grid generation. Finally, ligand docking was performed using Glide in the standard precision (SP) mode, with flexible ligand sampling. The following parameters were applied: a partial charge cutoff of 0.15, a van der Waals radius scaling factor of 0.80, and a reward for intramolecular hydrogen bond formation.^35^ For each ligand, the top-ranked pose based on the Glide docking score was selected as the final predicted pose.

#### 2.1.3 Molecular Docking with AutoDock Vina

Each protein target was first processed using AutoDockTools v1.5.7 following standard protocols and then saved in PDBQT format.^36^ The reference cocrystallized ligands provided by the challenge were used to define the center of the docking grid. Ligand preparation was carried out using the prepare_ligand4.py script from MGLTools v1.5.7,^37^ which converted each test set ligand into PDBQT format. Docking was then performed using AutoDock Vina v1.1.2,^36^ with each ligand docked to its corresponding protein target. For each ligand, the top-ranked pose based on the lowest predicted binding affinity was selected as the final predicted pose.

#### 2.1.4 Flexible Ligand Superposition with FlexS

Flexible ligand superposition was performed using FlexS v5.3.1.^11^ Since FlexS requires a rigid reference structure for pose prediction, we first conducted template selection using the 770 cocrystallized ligand poses available in the training set for SARS-CoV-2 Mpro. For each test set ligand, all 770 training set structures were considered as potential templates. The minimum similarity score threshold was set to 0.5. The template that yielded the highest similarity score was selected as the final template. Using this template, FlexS generated a set of predicted poses for the test ligand, and the pose with the highest FlexS ranking score was chosen as the final predicted pose.

#### 2.1.5 Deep Learning-Based Modeling with AlphaFold3

Since both SARS-CoV-2 Mpro and MERS-CoV Mpro are homodimers, with ligands binding only to a single monomer, we used the amino acid sequence of chain A as the input for the protein component. The CXSMILES strings of all ligands in test set were converted to meet JSON formatting requirements, including the replacement of backslashes (\) with double backslashes (\\) and quotation marks (″) with escaped quotes (\″). To generate high-accurate protein–ligand complex models, we sampled 100 random seeds, with 5 models generated per seed, resulting in a total of 500 predicted complex structures per ligand. The model with the highest AlphaFold3 internal ranking score among the 500 candidates was selected as the final predicted pose.

#### 2.1.6 Deep Learning-Based Modeling with DiffDock

For each ligand in test set, the receptor structure in PDB format and the ligand in SMILES format were provided as input. Inference was performed using the default configuration file from the official DiffDock implementation (https://github.com/gcorso/DiffDock/tree/main). Multiple candidate poses were generated per ligand, ranked according to DiffDock’s internal confidence score, and the top-scoring pose was selected as the final prediction.

#### 2.1.7 Deep Learning-Based Modeling with Boltz-2

Boltz-2 was applied for pose generation using only the amino acid sequence of the target protein, with multiple sequence alignment (MSA) obtained through the official Boltz-2 MSA server.^38^ Test set ligands were provided in SMILES format. For each ligand–protein pair, inference was performed with the pretrained Boltz-2 model using a diffusion sampling parameter of 10. The generated poses were ranked by Boltz-2’s internal confidence score, and the top-ranked pose was selected as the final prediction.

#### 2.1.8 Deep Learning-Based Modeling with Gnina

Pose prediction with Gnina was performed under four protocols to evaluate the effects of receptor flexibility and postdocking minimization: (i) flexible receptor, where side chains within 3.5 Å of the binding pocket were treated as flexible; (ii) flexible receptor with minimization, where generated poses were further refined using Gnina’s built-in minimization; (iii) rigid receptor, with the receptor kept fixed; and (iv) rigid receptor with Gnina’s built-in minimization. Ligands were provided in SDF format, and receptor structures in PDB format. Candidate poses were ranked using Gnina’s convolutional neural network (CNN) scoring function, and the top-ranked pose was selected as the final prediction.

All the predicted poses using the above protocols were reviewed to remove only physically impossible geometries.

### 2.2 Potency Prediction

#### 2.2.1 Data Preparation

We used the data set provided by the ASAP Discovery Antiviral Potency Prediction Challenge 2025, hosted on the Polaris platform.^4^ Access to the data set required authentication via the Polaris CLI (polaris login), followed by programmatic access using the polaris Python API.^6^

For each protein target, the data set comprised 1031 compounds in the training set and 297 compounds in the test set. Each data point included a comprehensive set of molecular descriptors along with experimentally determined potency values, as summarized in Table S2. To focus on biologically relevant compounds while retaining a sufficiently large data set for training, we excluded ligands with pIC_50_ values below 4, as those compounds exhibit weak or negligible activity and may introduce noise or bias into the modeling process. After filtering, 837 and 842 ligands remained for MERS-CoV Mpro and SARS-CoV-2 Mpro in the training set, respectively. Since all ligand structures were provided in CXSMILES format, we generated 3D conformations by converting each CXSMILES string into a MOL2 file using Open Babel v3.1.0.^28^ Then, all the input ligands were visually checked for chemical validity before modeling.

#### 2.2.2 LRIP-SF

In our previous work, we developed a machine learning-based scoring function called the ligand–residue interaction profile scoring function (LRIP-SF), which predicts ligand binding affinities by leveraging detailed ligand–residue interaction profiles.^25,39^ We first applied the LRIP-SF pipeline to the training set to identify the best-performing machine learning algorithm. This best performed model was then used to predict binding affinities for the test set ligands. The overall LRIP-SF workflow is illustrated in Figure 1 and described in detail below.

**Figure 1:**
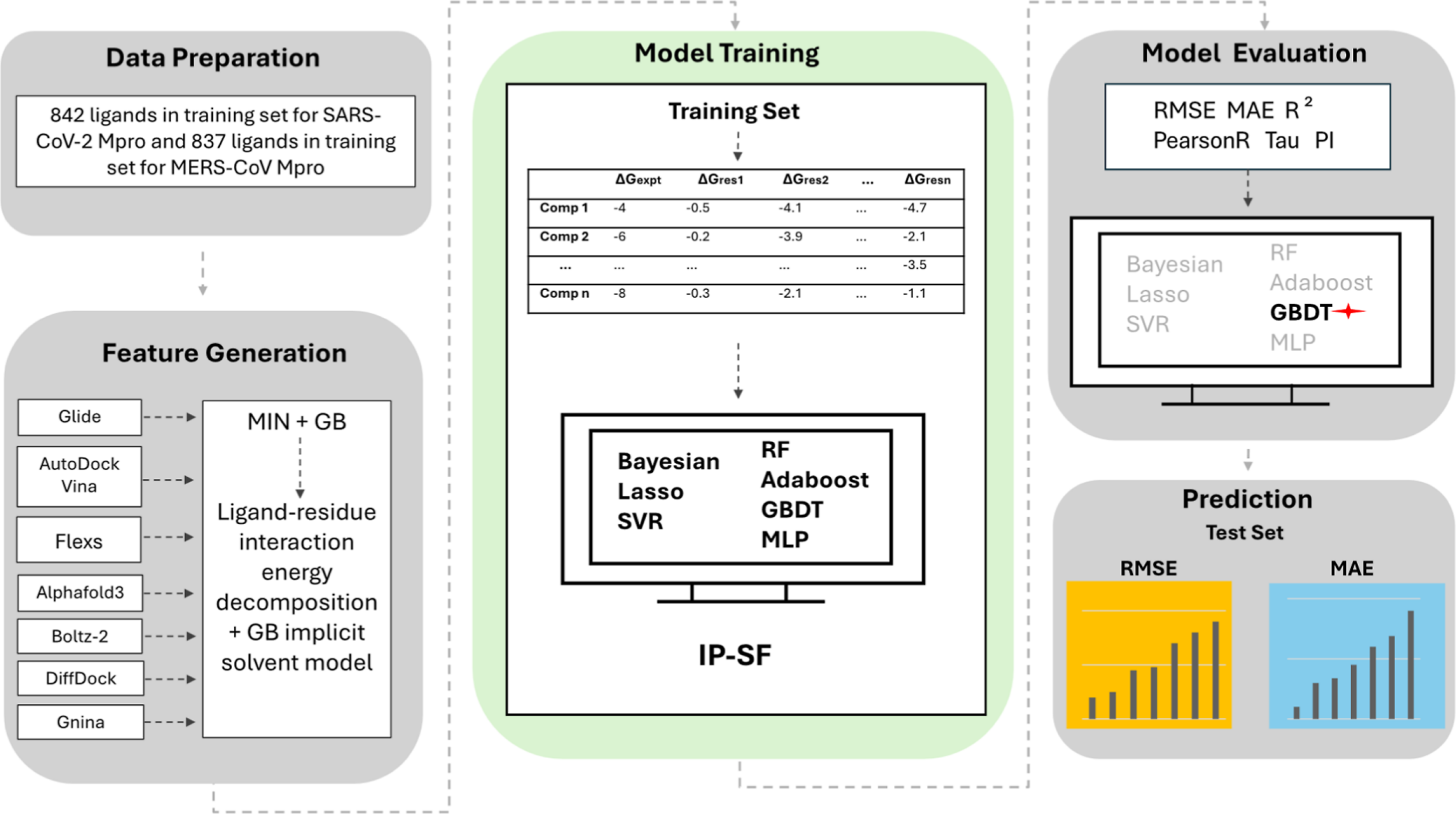
Workflow of the ligand–residue interaction profile scoring function (LRIP-SF) pipeline. The pipeline consists of pose generation using seven methods (Glide, AutoDock Vina, FlexS, AlphaFold3, Boltz-2, DiffDock and Gnina), energy minimization, MM-GBSA free energy decomposition and LRIP generation. Then the resulting LRIP are used as input features to train machine learning models for binding affinity prediction.

##### 2.2.2.1 Pose Generation

The first step in using LRIP-SF to predict binding affinity was pose generation. We employed the same three strategies used in ligand pose prediction subchallenge for each ligand: molecular docking with Glide and AutoDock Vina, flexible ligand superposition with FlexS, deep learning-based modeling with AlphaFold3, Boltz-2, DiffDock and Gnina. For Gnina, we adopted the Rigid Receptor without minimization protocol, which demonstrated the highest performance among the four tested protocols in the pose prediction subchallenge. All the predicted poses were reviewed to remove only physically impossible geometries.

We used the same reference structures for binding pose prediction to generate docking grid files and applied the same docking protocols to produce binding poses. For FlexS, unlike the binding pose prediction challenge for which all the crystal ligands could serve as the templates for matching, only the reference structure of a drug target was used as the template. For AlphaFold3-based pose generation, we used the amino acid sequence of chain A and the ligand’s CXSMILES string as input. The protonation states of the predicted ligand poses were subsequently assigned using the Maestro Epik module^35^ at default pH value of 7.0 ± 2.0.

##### 2.2.2.2 Molecular Mechanics Minimization and Ligand–Residue Free Energy Decomposition

We applied energy minimization using the GBSA implicit solvent model,^40,41^ which provided a good trade-off between accuracy and efficiency. Proteins were treated with the AMBER FF14SB force field,^42^ and ligands were treated with GAFF2 force field^43^ and assigned ABCG2 charges^44,45^ computed via the SQM module in AMBER 24.^46,47^ Each structural relaxation consisted of five sequential position-restrained minimizations, with the harmonic restraints on the protein and ligand being progressively relaxed. Specifically, for the protein, restraint force constants were applied in the order of 10, 5, 2, 1, and 0 kcal/mol/Å^2^; for the ligand they were 100, 50, 20, 10, and 0 kcal/mol/Å^2^. Each minimization consisted of 1000 steps, beginning with 200 steps of steepest descent followed by 800 steps of conjugate gradient optimization. All minimizations were performed using the PMEMD.cuda module in AMBER 24.

Ligand–residue interaction energies were computed using the same GBSA model during minimization for the final minimized structures. Energy decomposition was carried out with the SANDER module of AMBER 24.

##### 2.2.2.3 Machine Learning Model and Binding Affinity Prediction

We evaluated seven machine learning algorithms for binding affinity prediction: Lasso regression, Bayesian regression, support vector regression (SVR), random forest (RF), adaptive boosting (Adaboost), gradient-boosted decision trees (GBDT) and multilayer perceptron (MLP). Except for MLP which was implemented using the Keras module, all the other ML algorithms were implemented using the scikit-learn package in Python. Hyperparameters were kept at their default values in the respective libraries, and no large-scale hyperparameter tuning (such as grid search or nested cross-validation) was performed.

The input features for each model consisted of the ligand–residue interaction energy profiles. Prior to training, we filtered out insignificant features (residue–ligand pairs with minimum interaction energies greater than −0.1 kcal/mol) to reduce noise and improve model robustness. We employed bootstrap sampling to assess model stability. For each model, we conducted ten bootstrap replicates, with each run randomly splitting the data into 80% training and 20% validation sets.

To evaluate model performance, we calculated seven statistical metrics: root mean squared error (RMSE), mean absolute error (MAE), Pearson correlation coefficient (Pearson *R*), coefficient of determination (*R* ^2^), *p*-value, prediction index (PI) and Kendall’s tau rank correlation coefficient (TAU). The metrics values were calculated using sklearn.metrics and scipy.stats modules in Python. Based on these evaluation results, the best-performing model was selected for predicting the binding affinity of the test set ligands.

After final predictions were made, we evaluated the discrimination performance of different methods using receiver operating characteristic (ROC) curves and quantified it by the area under the curve (AUC). To provide a complementary assessment of early enrichment, we also computed enrichment factor (EF) values at various screening depths (1%, 5%, 10%, 20%, and 40%) along with the corresponding Top_1%, Top_5%, Top_10%, Top_20%, and Top_40% hit rates.

#### 2.2.3 Global Sensitivity Analysis (GSA)

GSA^26^ was performed by systematically removing one feature at a time and evaluating the resulting increase in RMSE compared to the baseline model using the full feature set. To account for potential synergistic effects among residues involved in ligand binding, we grouped residues with Pearson’s correlation coefficients greater than 0.85 and treated a group as a single feature during the analysis.

## 3 Results

### 3.1 Structures of SARS-CoV-2 Mpro and MERS-CoV Mpro

SARS-CoV-2 Mpro and MERS-CoV Mpro share a highly conserved overall architecture, as illustrated in Figure 2. Both proteases function as homodimers (Figure 2A,C), and each monomer comprises three domains: domain I, domain II, and domain III (Figure 2B,D). Domains I and II form an antiparallel β-barrel structure, creating a cleft that serves as the substrate-binding site. Domain III, composed of α-helices, facilitates dimerization through intermonomer interactions.^2^ To assess structural similarity more precisely, we performed a structural alignment of chain A from each protein (Figure 2E), which revealed a high degree of overlap, particularly in the binding site region. Figure 2F highlighted the close spatial alignment of the cocrystallized ligands. This structural conservation suggested that the ligand-binding modes and key interaction features may also be shared across the two viral proteases.

**Figure 2:**
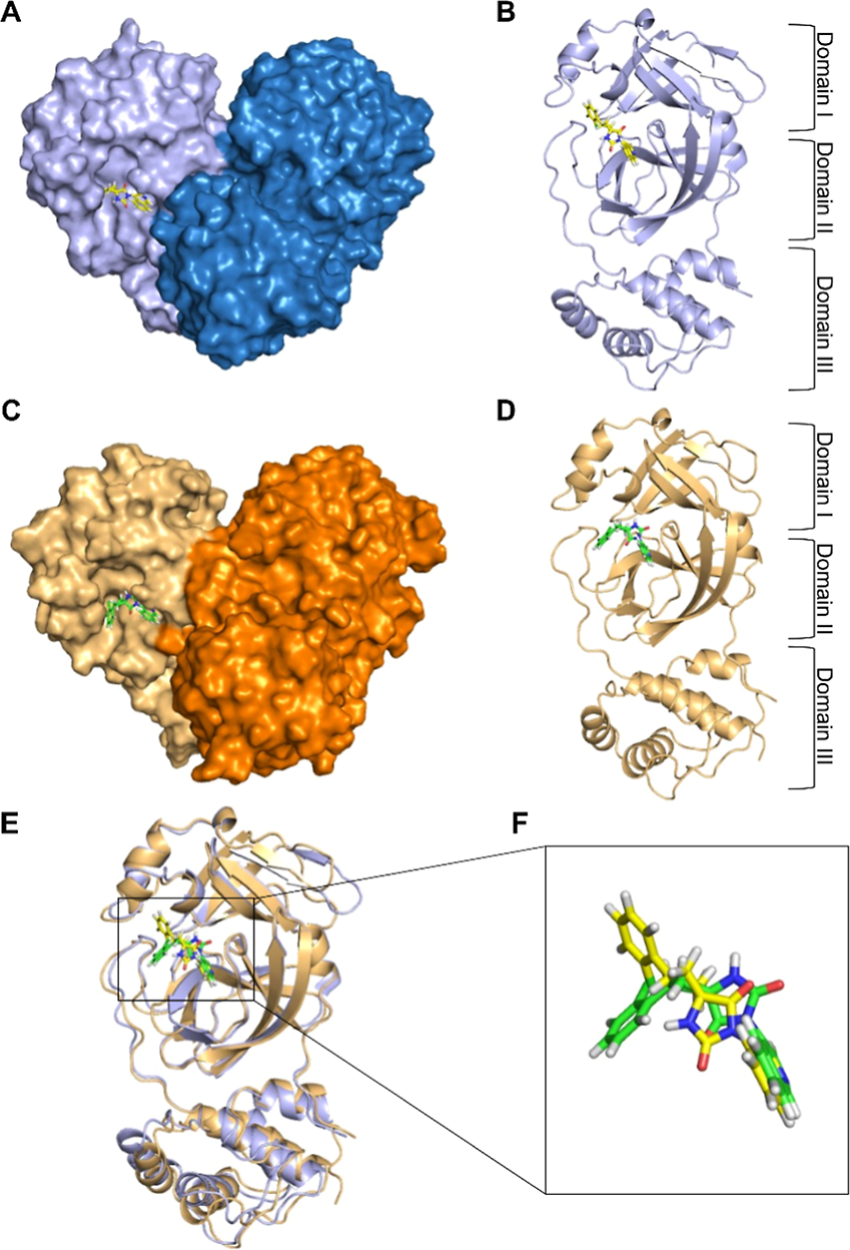
Structural comparison of SARS-CoV-2 Mpro and MERS-CoV Mpro. (A) Surface representation of SARS-CoV-2 Mpro homodimer. Protomers A and B are shown as purple and blue solid surfaces and the cocrystallized ligand as yellow sticks. (B) Cartoon representations of protomer A of SARS-CoV-2 Mpro with the cocrystallized ligand. (C) Surface representation of MERS-CoV Mpro homodimer. Protomers A and B are shown as light orange and dark orange solid surfaces and the cocrystallized ligand as green sticks. (D) Cartoon representations of protomer A of MERS-CoV Mpro with the cocrystallized ligand. (E) Structural alignment of protomer A from both main proteases. (F) Close-up view of the aligned ligands resided in the binding pocket.

### 3.2 Pose Prediction

Root-mean-square deviation (RMSD) was used as the primary evaluation metric. A predicted pose was considered successful if its heavy-atom RMSD relative to the crystallographic reference was less than 2.0 Å. Success rates were estimated using 1000 bootstrap replicates with replacement.

However, due to inconsistencies in chain orientation across the training set and test set, a preprocessing step was necessary. Although all crystallographic ligands in the test set bound to chain A, in some protein structures, chain A was located on the opposite side of the dimer compared to the others. This would lead to incorrect RMSD calculations if they were not corrected.

To address this, we performed structural alignment of the predicted complexes prior to RMSD evaluation. All alignment steps were carried out using the Antechamber module^48^ in AMBER 24.^46,47^ First, the protein structure from each predicted complex was aligned to its corresponding crystallographic protein structure. Next, atom names and atom sequence in each predicted ligand’s MOL2 file were adjusted to match those of the corresponding crystallographic ligand. Finally, each predicted ligand was transformed based on the resulting translation–rotation transformation matrix from receptor alignment, and RMSD was computed after the transformation directly without the least-squares fitting.

As shown in Figure 3, AlphaFold3 achieved the highest success rate (88.1 ± 2.4%), followed by Boltz-2 (84.1 ± 2.7%), FlexS (51.5 ± 3.7%), Glide (24.6 ± 3.2%), DiffDock (12.3 ± 2.3%), AutoDock Vina (3.1 ± 1.2%) and Gnina (1% to 3.1%). The corresponding RMSD distributions are presented in Figure 4, where the cumulative fraction of successful poses was plotted as a function of RMSD. AlphaFold3 showed the steepest curve, indicating a higher proportion of low-RMSD predictions across the test set, while Boltz-2 demonstrates comparably strong performance. Table 1 summarized the detailed performance metrics for each method, including average RMSD and their standard deviation, as well as the minimum and maximum observed RMSD values. For Gnina, the rigid receptor without minimization protocol achieved the highest performance among the four tested protocols. Therefore, only this protocol was selected for subsequent integration into the IPSF workflow. Top1, Top5 and Top10 success rates are also calculated and reported in Table S17.

**Figure 3:**
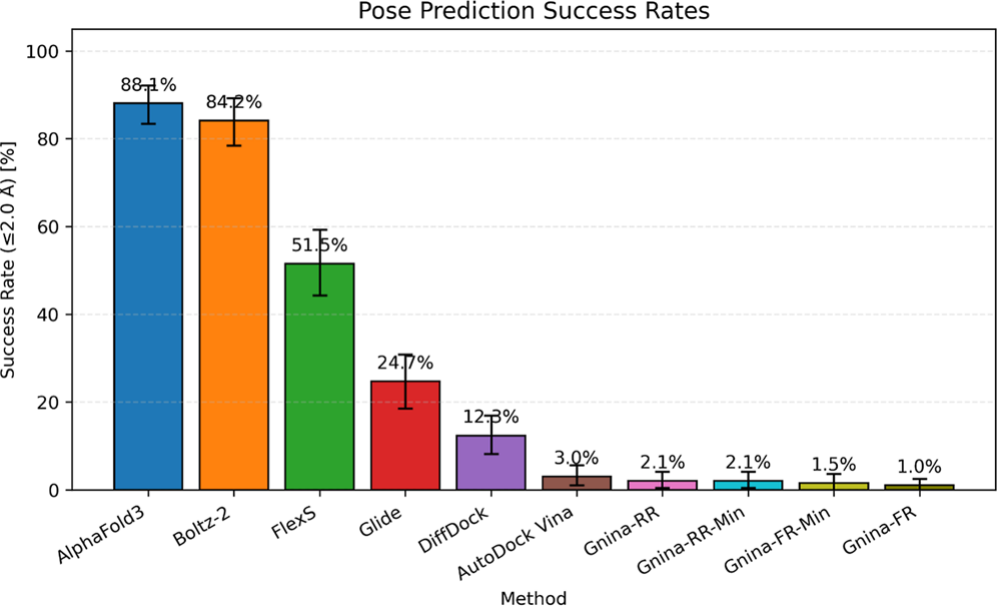
Success rate for each method, defined as the percentage of predicted ligand poses with RMSD less than 2 Å. Gnina-RR: Gnina rigid receptor protocol; Gnina-RR-Min: Gnina rigid receptor + minimization protocol; Gnina-FR: Gnina flexible receptor protocol; Gnina-FR-Min: Gnina flexible receptor + minimization protocol. Error bar indicates 95% confidence interval.

**Figure 4:**
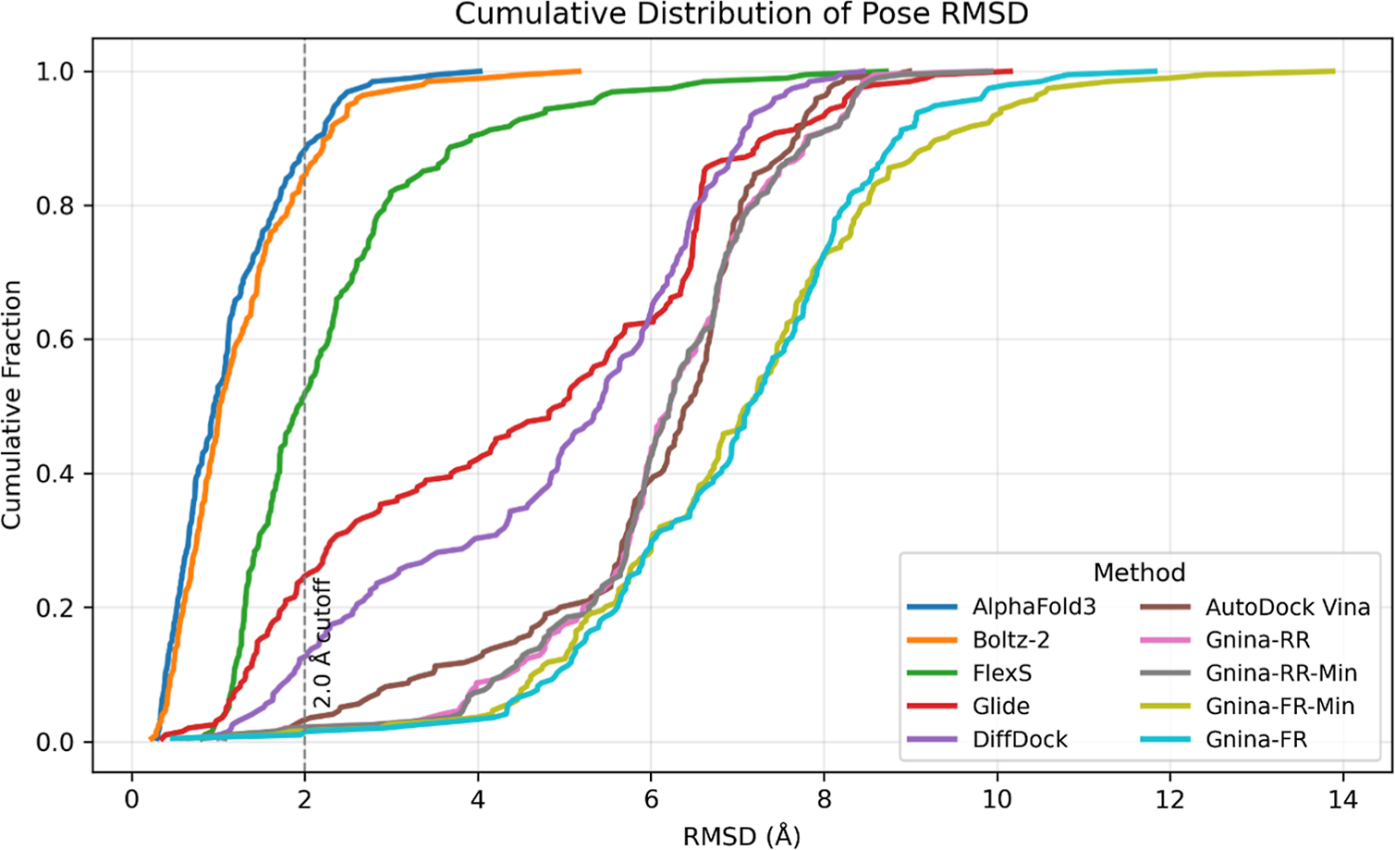
Cumulative distributions of RMSD values across all predicted poses for each method. Gnina-RR: Gnina rigid receptor protocol; Gnina-RR-Min: Gnina rigid receptor + minimization protocol; Gnina-FR: Gnina flexible receptor protocol; Gnina-FR-Min: Gnina flexible receptor + minimization protocol.

**Table 1:** Summary of Pose Prediction Performance

method	total ligands	success rate (<2 Å) (%)	mean RMSD (Å)	max RMSD (Å)	min RMSD (Å)
AlphaFold3	193	88.1 ± 2.4	1.122	4.024	0.293
Boltz-2	195	84.2 ± 2.7	1.251	5.167	0.233
FlexS	194	51.5 ± 3.7	2.333	8.718	0.806
Glide	195	24.7 ± 3.2	4.500	10.154	0.353
DiffDock	195	12.3 ± 2.3	4.887	8.451	0.669
AutoDock Vina	195	3.0 ± 1.2	6.002	8.989	0.828
Gnina-RR	194	2.1 ± 1.1	6.159	9.863	1.006
Gnina-RR-Min	194	2.1 ± 1.1	6.164	9.926	1.074
Gnina-FR-Min	194	1.5 ± 2.1	7.062	13.880	0.588
Gnina-FR	194	1.0 ± 1.5	6.991	11.823	0.475

### 3.3 Potency Prediction

We used seven evaluation metrics to assess the performance of machine learning models for both protein targets: root mean squared error (RMSE), mean absolute error (MAE), Pearson correlation coefficient (Pearson *R*), *p*-value, coefficient of determination (*R* ^2^), Kendall’s tau (TAU), and prediction index (PI). All metrics were computed using the sklearn.metrics and scipy.stats modules in Python, with results summarized in Tables S3–S16. Among the evaluated ML algorithms, the gradient boosting decision tree (GBDT) consistently outperformed others and was therefore selected for predicting the binding affinities of the test set ligands.

After making predictions with the GBDT model, we calculated the seven statistical metrics (RMSE, MAE, Pearson *R*, *R* ^2^, *p*-value, TAU, and PI) to evaluate the model performance on the test set. Some ligands were excluded from the final evaluation due to the following reasons: (1) FlexS failed to generate valid poses for some ligands that did not meet the similarity score threshold of 0.5; (2) AlphaFold3 produced chemically invalid poses for certain ligands; (3) some SARS-CoV-2 Mpro test ligands lacked experimental potency values; (4) A number of enantiomers occurred in both the train and test set. These molecules share the same SMILES but differ in the spatial arrangement of their atoms and have different experimental IC_50_ values.

It is noted that we additionally evaluated Boltz-2’s capability to directly predict binding affinity, independent of the LRIP-SF workflow. Also, for Gnina, only the Gnina-RR (rigid receptor without ligand minimization) was retained for the subsequent LRIP-SF construction, as it outperformed other Gnina protocols in the pose-prediction subchallenge. Furthermore, we tested Gnina’s scoring capability using the Top 1 ligand poses generated by AlphaFold3.

The final evaluation results were reported in Tables 2 and 3. Predicted potency values from all ligand pose generation methods demonstrated statistically significant correlations with experimental binding affinities, as measured by Pearson correlation coefficients (*R*) and their associated *p*-values. All Pearson correlations were statistically significant with *p* < 10^–15^, far surpassing conventional thresholds for significance (*p* < 0.05), indicating robust predictive performance. The comparison of prediction errors using different ligand pose generation protocols across two protein targets was shown in Figures 5 and 6. Overall AlphaFold3 achieved better performance, followed closely by Boltz-2 and FlexS. The 95% CI values for evaluation metrics are reported in Tables S22 and S23.

**Table 2:** Statistical Performance Metrics of Potency Prediction for MERS-CoV Mpro Using Different Ligand Pose Generation Protocols

method	MAE	RMSE	Pearson’s *R*	*p*-value	*R* ^2^	Kendall’s tau	PI
AlphaFold3	0.606 ± 0.032	0.813 ± 0.060	0.643 ± 0.050	3.51 × 10^–35^	0.340 ± 0.063	0.477 ± 0.030	0.739 ± 0.015
Boltz-2-Internal	1.470 ± 0.048	1.672 ± 0.055	0.696 ± 0.062	3.89 × 10^–43^	–1.805 ± 0.245	0.556 ± 0.027	0.779 ± 0.014
Boltz-2	0.658 ± 0.035	0.896 ± 0.068	0.538 ± 0.076	7.18 × 10^–23^	0.197 ± 0.082	0.447 ± 0.034	0.724 ± 0.017
DiffDock	0.676 ± 0.034	0.898 ± 0.060	0.575 ± 0.055	7.60 × 10^–27^	0.194 ± 0.063	0.446 ± 0.028	0.723 ± 0.014
FlexS	0.612 ± 0.033	0.828 ± 0.064	0.626 ± 0.053	2.27 × 10^–32^	0.309 ± 0.068	0.493 ± 0.028	0.747 ± 0.014
Glide	0.737 ± 0.037	0.961 ± 0.056	0.442 ± 0.057	1.81 × 10^–15^	0.078 ± 0.058	0.354 ± 0.031	0.677 ± 0.016
Gnina-RR	0.712 ± 0.035	0.929 ± 0.053	0.491 ± 0.050	7.02 × 10^–19^	0.135 ± 0.054	0.380 ± 0.030	0.690 ± 0.015
Gnina-Scoring	2.178 ± 0.111	2.834 ± 0.154	0.216 ± 0.072	2.03 × 10^–4^	–7.049 ± 0.819	0.220 ± 0.040	0.610 ± 0.020
AutoDock Vina	0.681 ± 0.032	0.873 ± 0.050	0.588 ± 0.047	2.74 × 10^–28^	0.239 ± 0.054	0.475 ± 0.028	0.738 ± 0.014

**Table 3:** Statistical Performance Metrics of Potency Prediction for SARS-CoV-2 Mpro Using Different Ligand Pose Generation Protocols

method	MAE	RMSE	Pearson’s *R*	*p*-value	*R* ^2^	Kendall’s tau	PI
AlphaFold3	0.724 ± 0.033	0.894 ± 0.038	0.820 ± 0.026	1.12 × 10^–63^	0.548 ± 0.037	0.607 ± 0.026	0.804 ± 0.013
Boltz-2-Internal	0.950 ± 0.038	1.133 ± 0.051	0.845 ± 0.022	4.36 × 10^–71^	0.272 ± 0.072	0.638 ± 0.023	0.819 ± 0.012
Boltz-2	0.716 ± 0.036	0.909 ± 0.043	0.800 ± 0.027	9.54 × 10^–59^	0.532 ± 0.043	0.598 ± 0.024	0.799 ± 0.012
DiffDock	0.973 ± 0.044	1.192 ± 0.045	0.695 ± 0.037	9.93 × 10^–39^	0.195 ± 0.061	0.512 ± 0.028	0.756 ± 0.014
FlexS	0.722 ± 0.036	0.925 ± 0.045	0.782 ± 0.023	7.84 × 10^–53^	0.510 ± 0.045	0.587 ± 0.023	0.794 ± 0.012
Glide	0.860 ± 0.041	1.070 ± 0.046	0.724 ± 0.027	2.46 × 10^–43^	0.352 ± 0.055	0.524 ± 0.026	0.762 ± 0.013
Gnina-RR	1.000 ± 0.048	1.253 ± 0.052	0.638 ± 0.036	1.21 × 10^–30^	0.101 ± 0.074	0.450 ± 0.028	0.725 ± 0.014
Gnina-Scoring	4.190 ± 0.342	6.871 ± 0.594	0.350 ± 0.041	1.03 × 10^–8^	–25.954 ± 5.130	0.414 ± 0.031	0.707 ± 0.015
AutoDock Vina	0.862 ± 0.039	1.067 ± 0.044	0.718 ± 0.031	6.89 × 10^–42^	0.355 ± 0.055	0.531 ± 0.026	0.766 ± 0.013

**Figure 5:**
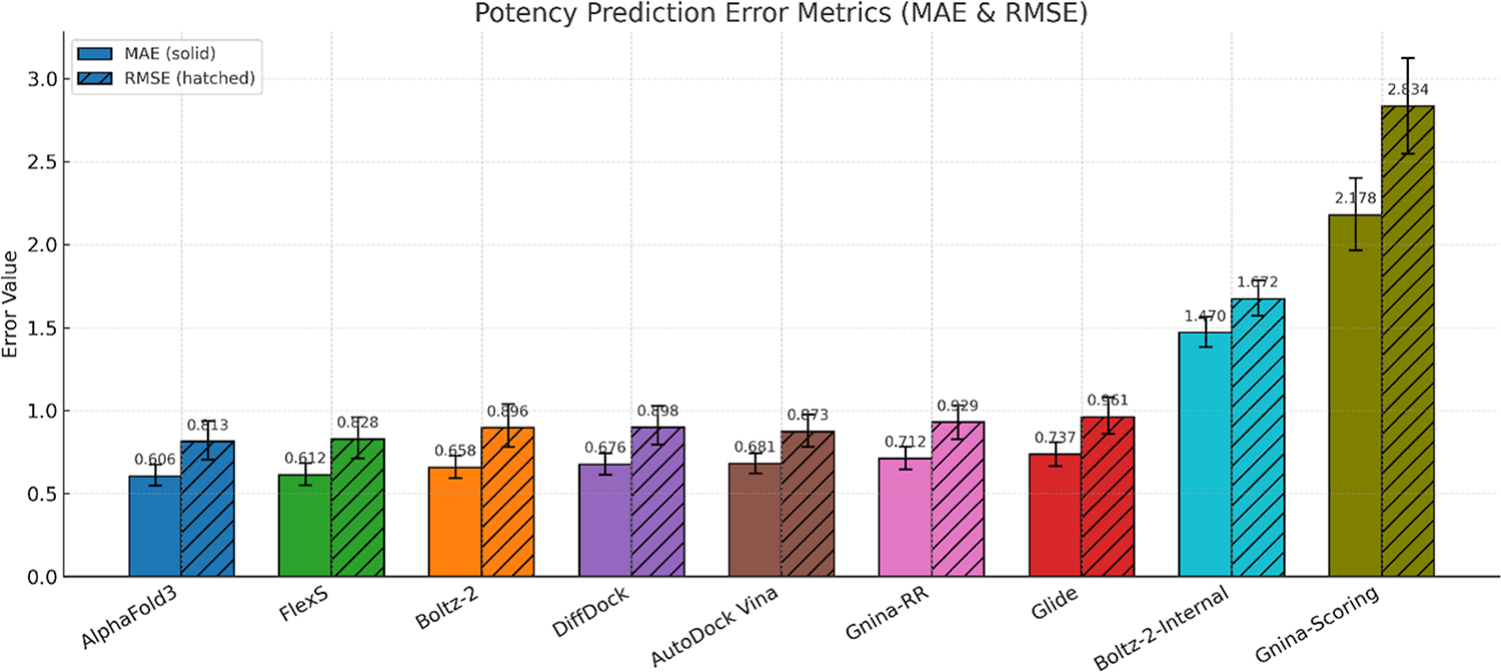
Comparison of mean absolute error (MAE) and root mean squared error (RMSE) for LRIP-SFs using different ligand pose generation protocols for MERS-CoV Mpro. Error bar indicated 95% confidence interval.

**Figure 6:**
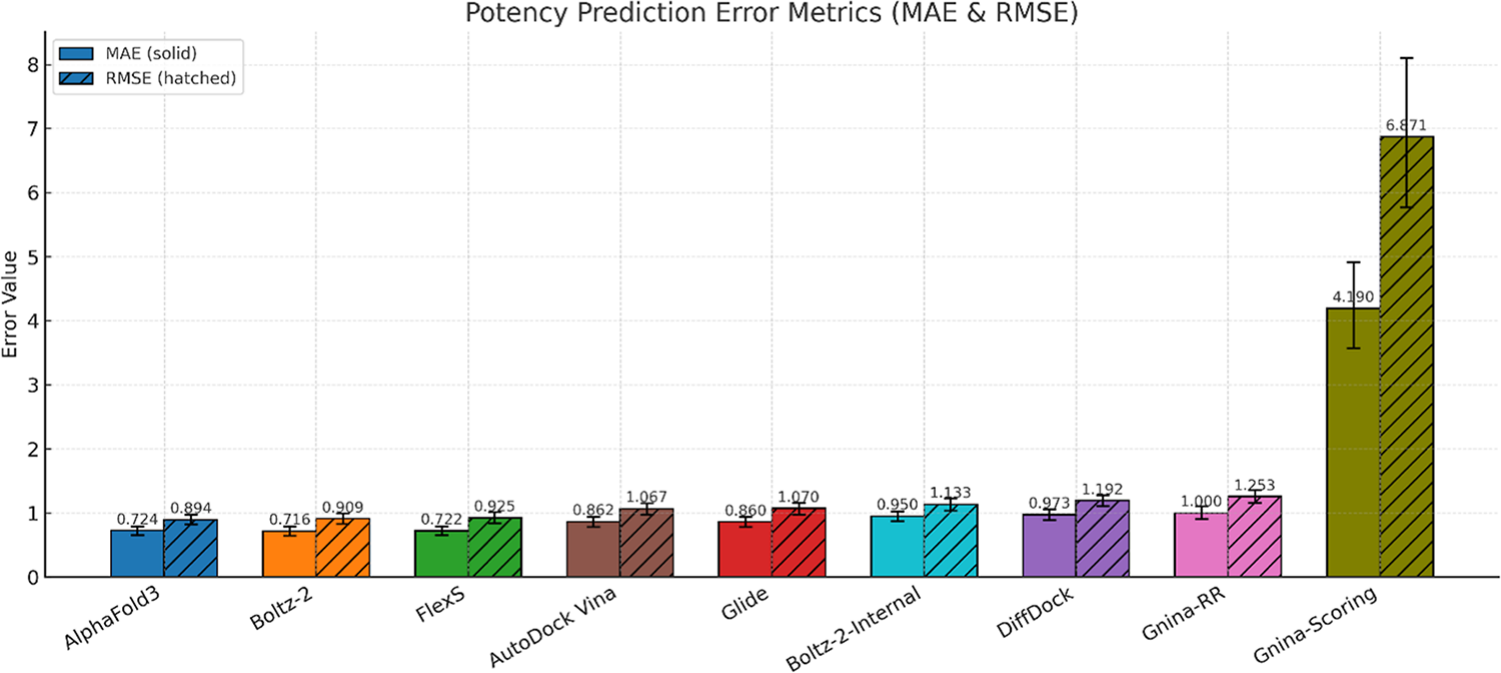
Comparison of mean absolute error (MAE) and root mean squared error (RMSE) for LRIP-SFs using different ligand pose generation protocols for SARS-CoV-2 Mpro. Error bar indicates 95% confidence interval.

To quantitatively assess whether the observed performance differences were statistically meaningful, we conducted a statistical significance analysis of the MAE and RMSE differences between AlphaFold3 and all other methods using 1000 bootstrap resamples. For each comparison, we evaluated significance using paired *t* tests. Across both the MERS-CoV and SARS-CoV-2 Mpro data sets, AlphaFold3 consistently achieved significantly lower MAE and RMSE values than the other methods (Tables S27–S30), with paired *t*-test *p*-values <0.05 in all but one comparison (MAE of AlphaFold3 versus FlexS on SARS-CoV-2 Mpro). In that specific case, the RMSE difference remained significant and AlphaFold3 produced a higher Pearson correlation coefficient, still indicating better prediction performance. Overall, these statistical results confirmed that AlphaFold3 achieved the best potency prediction performance when combined with LRIP-SF among the evaluated pose-generation approaches.

Further, the discrimination performance of each scoring or pose-prediction method is demonstrated in ROC curves in Figure 7. For both protein targets, most methods achieved AUC values well above AUC value of 0.8, indicating substantial ability to distinguish active from inactive compounds. For the MERS-CoV Mpro data set (Figure 7 left), Boltz-2-Internal prediction achieved the highest AUC (0.883), followed closely by AlphaFold3 (0.880), Vina (0.877), and FlexS (0.871). DiffDock, Boltz-2, and Gnina-RR showed intermediate performance (0.836), while Glide and Gnina-Scoring were comparatively lower (AUC = 0.786 and 0.687, respectively). For the SARS-CoV-2 Mpro data set (Figure 7 right), Boltz-2-Internal prediction again showing the best performance (AUC = 0.955), followed by FlexS (0.937), AlphaFold3 (0.924), and Boltz-2 (0.919). Glide, DiffDock, and Gnina-RR performed moderately (AUC ≈ 0.86–0.90), whereas Gnina-Scoring remained the least discriminative (AUC = 0.826).

**Figure 7:**
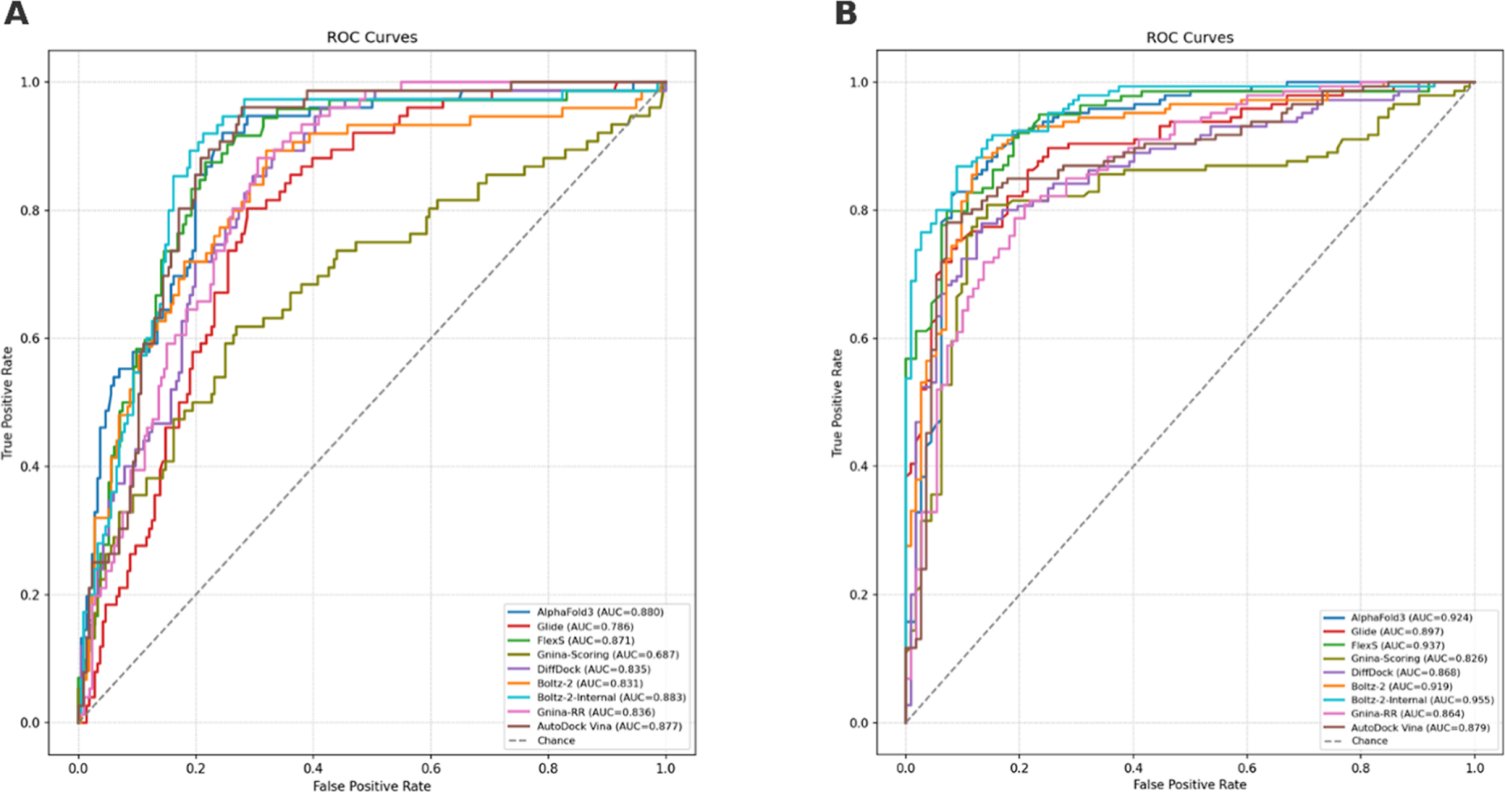
Comparative ROC analysis of different methods for hit classification. (A) ROC curves for MERS-CoV Mpro. (B) ROC curves for SARS-CoV-2 Mpro. Each curve shows the true positive rate versus the false positive rate for a given method, with the corresponding area under the curve (AUC) indicated in parentheses. The dashed diagonal line represents random-classification performance.

Collectively, these results demonstrate that the integrated LRIP-SF framework effectively leverages diverse docking and deep learning-based methods to achieve robust global discrimination across both viral protease systems. Early enrichment statistics (EF and hit rate at 1%, 5%, 10%, 20% and 40%) supporting these ROC-AUC are summarized in Tables S18–S21.

We further evaluated cross-target consistency by analyzing ligands for which AlphaFold3 generated poses for both MERS-CoV Mpro and SARS-CoV-2 Mpro. For each ligand, we compared the predicted and experimental differences in pIC_50_ (Δ_Pred vs Δ_Exp). As shown in Figure S1, Δ_Pred correlated with Δ_Exp (*y* = 0.66*x* – 0.39, *R* ^2^ = 0.29) with an RMSE of 0.64. Notably, 84% of ligands showed the correct direction of affinity change (*p* < 10^–28^, sign test), indicating that our workflow can reasonably capture relative potency trends across related proteases.

Another big advantage of LRIP-SF lies that it can be applied to decipher the underlying binding mechanism and identify hotspot residues. Global sensitivity analysis (GSA) was then performed for both protein targets to this end. Residues with similar ligand–residue interaction patterns were identified with the Pearson correlation coefficient being greater than 0.85. Those residue groups, listed in Tables S24 and S25, participated in GSA as a single feature set. For SARS-CoV-2 Mpro, the top five key residues or residue groups identified by GSA were H41, T135, Q19–N119, A191, I43–C44–T45–D48–M49–H164. For MERS-CoV Mpro, the top five key residues or residue groups are C148, P39–G177, F143, Q19, and P52. The resulting RMSEs upon deletion compared to the baseline were shown in Figure 8. Top-ranking residues and key residues shared by both Mpros around the binding site were highlighted by red boxes in Figure 9. Sequence alignment of protomer A from each Mpro was shown in Figure 9A. Figure 9B,C displays a test-set ligand (molecule ID: 1210) with high binding affinity for both Mpros, residing in binding pocket (pIC_50_ = 7.40 for MERS-CoV Mpro; pIC_50_ = 8.64 for SARS-CoV-2 Mpro). The complex structures were generated using AlphaFold3.

**Figure 8:**
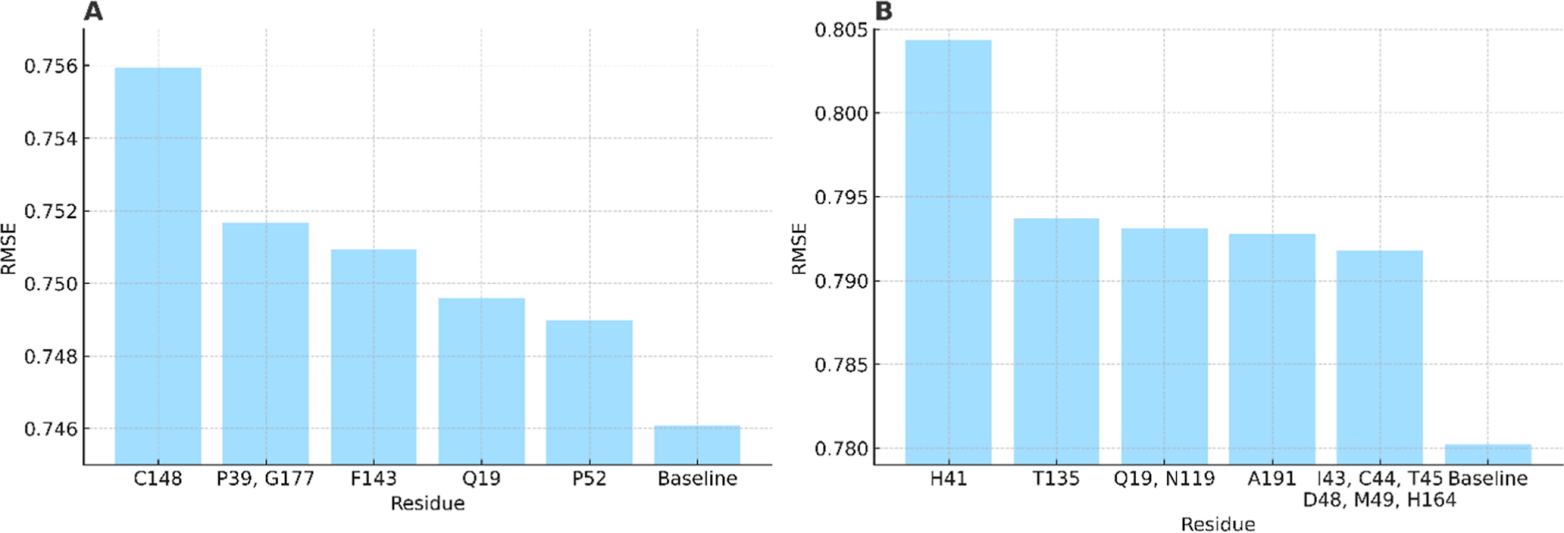
Global sensitivity analysis (GSA) results showing the impact of top-ranked residue groups on ligand binding for both viral proteases. (A) Residue groups important for MERS-CoV Mpro. (B) Residue groups important for SARS-CoV-2 Mpro.

**Figure 9:**
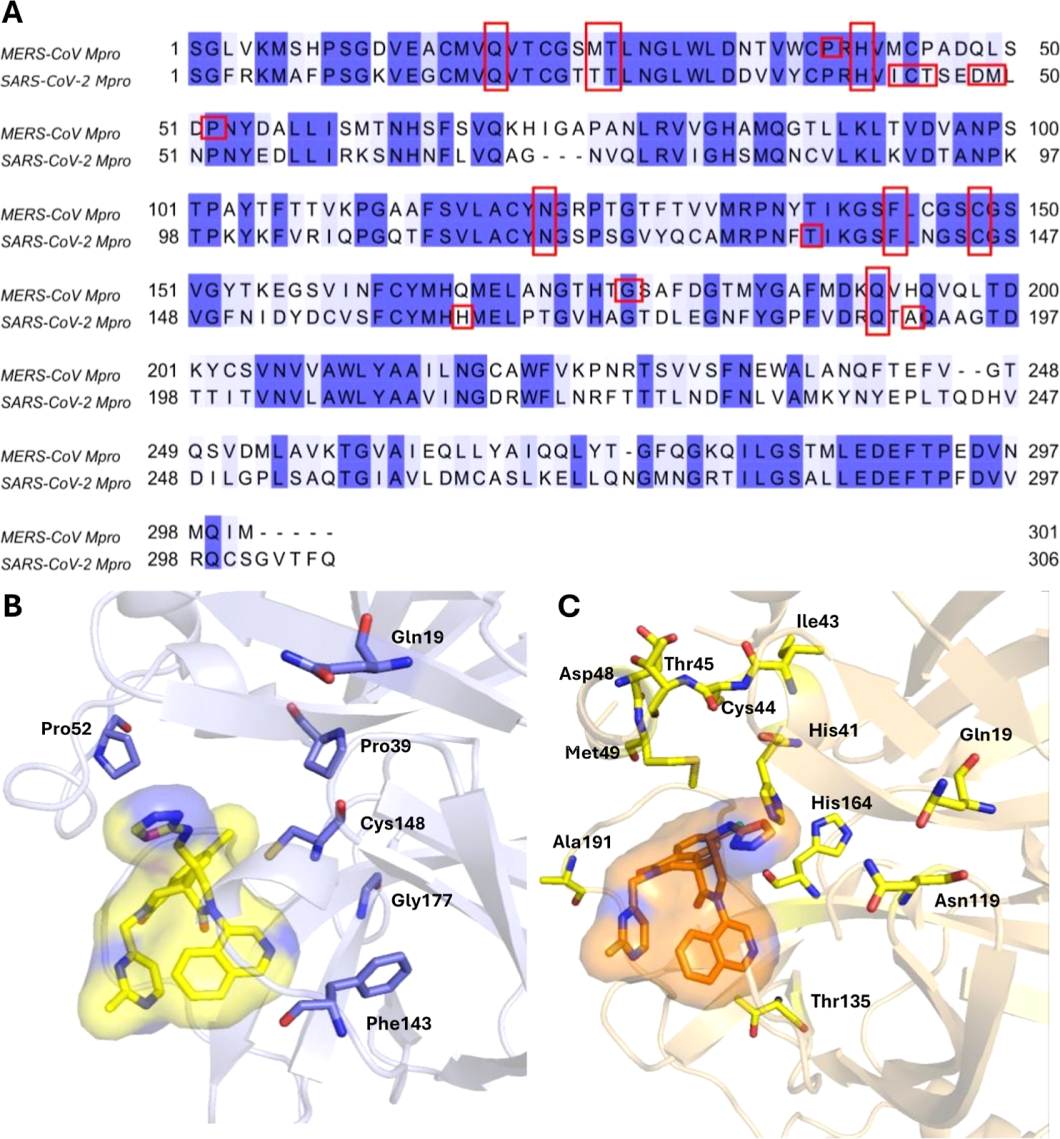
Comparative analysis of binding site conservation and ligand interaction across MERS-CoV Mpro and SARS-CoV-2 Mpro. (A) Sequence alignment of protomer A from MERS-CoV Mpro and SARS-CoV-2 Mpro. Conserved residues are shaded in blue, and key residues around the binding site are highlighted by red boxes. (B) Structure of MERS-CoV Mpro bound to a potent test-set ligand (molecule ID: 1210), modeled with AlphaFold3. The ligand is shown in yellow sticks and surface representation. Key residues identified by GSA are shown as purple sticks. (C) Structure of SARS-CoV-2 Mpro bound to a potent test-set ligand (molecule ID: 1210), modeled with AlphaFold3. The ligand is shown in orange sticks and surface representation. Key residues identified by GSA are shown as yellow sticks.

## 4 Discussion

In this retrospective analysis of the ASAP Antiviral Challenge 2025, our benchmarking results show that deep learning-based modeling with AlphaFold3 achieved the highest overall performance across both the pose and potency prediction subchallenges for MERS-CoV Mpro and SARS-CoV-2 Mpro. Boltz-2 also demonstrated performance comparable to AlphaFold3 in binding pose prediction and, when combined with LRIP-SF, in binding affinity prediction. Compared with traditional docking methods such as Glide and AutoDock Vina, which rely on predefined scoring functions and sampling algorithms, deep learning-based approaches more effectively captured complex protein–ligand interaction patterns, likely contributing to their superior accuracy. Unlike the small-ligand superposition method FlexS, which depends on structural similarity to known ligands, deep learning-based modeling offers greater flexibility and generalizability by de novo predicting poses without requiring pre-existing templates. These findings underscore the advantages of deep learning-based approaches over conventional docking methods, particularly for drug targets with limited structural characterization.

However, a key limitation of deep learning-based modeling, exemplified by AlphaFold3, is its relatively high computational cost. Table S26 summarizes the runtime and hardware configurations for each protocol. While AlphaFold3 and Boltz-2 achieved high predictive performance, they required substantially longer runtime than other methods. This considerable computational burden limits the practicality of such deep learning models for large-scale virtual screening campaigns, where thousands of compounds must be evaluated within a reasonable time frame.

In contrast, FlexS provided a favorable balance between computational efficiency and predictive accuracy in the challenge tasks. Despite its speed, FlexS remains constrained by its template-based nature. It aligns ligands solely based on structural similarity and does not account for the surrounding receptor environment, which may lead to biologically irrelevant poses or steric clashes with receptor residues. For the pose prediction subchallenge, we further tested an alternative strategy using only the reference ligand structure as the template instead of searching for the best match among all 770 training-set ligands. Under this condition, the success rate decreased to 22.5% with an average RMSD of 3.395 Å. Although slightly lower than Glide’s success rate (24.7%), FlexS still achieved a smaller average RMSD (3.4 Å vs 4.5 Å), indicating competitive accuracy. These findings suggest that the performance of FlexS is influenced by the availability and quality of suitable template ligands, and that even with minimal template selection, it can still produce reasonably accurate poses. Future improvements like incorporating receptor information into the ligand-superposition process could reduce potential steric clashes and improve biological relevance, further enhancing its success in structure-based drug design.

Regarding potency prediction, we also noticed that AlphaFold3 would provide a ranking score along with its predictions. It is interesting to investigate if AlphaFold3 internal ranking score can serve as a predictor of binding potency. We further evaluated the correlation between AlphaFold3 internal ranking score and experimentally determined binding affinity. The results were shown in Figure S2. For MERS-CoV Mpro, the linear regression equation is *y* = −8.114*x* + 0.950 (*R* = 0.12), and for SARS-CoV-2 Mpro, it is *y* = −14.004*x* + 5.864 (*R* = 0.17), indicating a weak linear relationship between AlphaFold3 ranking score and ligand potency. Although the higher the AlphaFold3 ranking score, the higher the potency is for both main proteases, the correlation is too weak to merit AlphaFold3 ranking score as a good predictor of potency for the two proteases.

In the ASAP Antiviral Challenge 2025, other participating teams also employed diverse and advanced strategies to enhance predictive performance.

For pose prediction subchallenge, the highest-performing method utilized a diffusion-based generative model that refined ligand poses through iterative updates of atomic coordinates, beginning from an initial rough guess and converging on accurate binding conformations.^49^ This approach effectively captured subtle protein–ligand interactions and resulted in a success rate of 86.378 ± 2.466% for poses with RMSD below 2 Å, with an average RMSD of 1.330 ± 0.266 Å.^50^ Another leading team integrated a customized AlphaFold3-like architecture trained on SARS-CoV-2 and MERS-CoV Mpro data with template-based docking using ClusPro LigTBM.^50^ Ligand poses were generated through substructure matching with known templates, followed by physics-based sampling, energy minimization, or torsional diffusion. A density-based filter and confidence scoring were applied to select final poses. This method achieved a success rate of 85.213 ± 2.601% and an average RMSD of 1.388 ± 0.267 Å.^50^ A third high-ranking approach combined a fine-tuned Boltz-1 model with the diffusion-based DiffDock-L framework.^51^ Boltz-1 was trained on SARS-CoV-2 Mpro complexes to improve protein–ligand structural prediction, and the resulting structures served as priors for ligand pose refinement using DiffDock-L.^51^ This two-stage workflow focused on optimizing RMSD and produced a success rate of 83.657 ± 2.769% with an average RMSD of 1.549 ± 0.269 Å.^50^

For potency prediction subchallenge, one top-performing method, MolE,^52^ is a transformer-based model tailored for molecular graphs, featuring a two-step pretraining scheme: self-supervised learning on ∼842 million unlabeled molecules to capture chemical structure, followed by multitask learning to incorporate biological context. This approach achieved an aggregated MAE of 0.509 ± 0.022 across the two protein targets.^50^ Another high-ranking team evaluated several model architectures, including MolE, MolGPS, and models from the MolFlux package, ultimately selecting MolGPS-based models for each end point based on cross-validation performance.^53^ This strategy yielded an aggregated MAE of 0.516 ± 0.022.^50^ A third top-performing team developed an ensemble ML predictor leveraging fingerprints, physicochemical properties, molecular graphs, and SMILES-based large language models.^54^ They trained five types of base learners (RF, LGBM, MLP, GNN, and LLM) with three replicates each, combining their outputs for enhanced robustness, achieving an aggregated MAE of 0.517 ± 0.021.^50^

While the best-performing submissions in this challenge reported mean absolute errors (MAE) around 0.5 in pIC_50_ units, our results of approximately 0.6–0.7 remain close to this level and demonstrate a comparable degree of predictive reliability. It is important to emphasize that the typical experimental uncertainty associated with biochemical *K*_i_ or IC_50_ measurements is already on the order of 0.3–0.5 kcal/mol in free energy units,^55,56^ depending on assay format, replicate conditions, and data normalization procedures. Within this context, the observed performance gap of roughly 0.2 kcal/mol between our model and the top submissions is modest. Therefore, despite not achieving the absolute lowest MAE or RMSE reported in the challenge, our model can be considered to perform near the state-of-the-art level in practical terms.

Altogether, our benchmarking results highlight how the increasing integration of deep learning, physics-based modeling, and ligand superposition techniques is reshaping structure-based drug discovery. In addition to the comparative performance analysis, this study also highlights three methodological aspects that extend beyond prior evaluations. First, incorporating AlphaFold3-generated ligand poses into the physics-based LRIP-SF workflow yields the best binding affinity prediction performance, underscoring the critical role of high-quality initial poses. Second, shape-based ligand superposition method offers comparable accuracy in pose prediction and potency prediction when embedded within the LRIP-SF workflow, while being much more time-efficient than AlphaFold3 in binding pose prediction. Third, the application of global sensitivity analysis provides residue-level interpretability of predicted binding affinity and reveals which interaction features contribute most to ligand binding. Collectively, these insights can guide the adoption of optimal strategies for protein–ligand binding affinity prediction.

While computational cost remains a practical constraint, continued advances in model efficiency, hardware acceleration, and hybrid workflows are expected to make accurate, large-scale structure prediction increasingly accessible. These developments will accelerate the discovery of antiviral therapeutics by enabling more reliable and efficient integration of pose prediction, binding-affinity estimation, and data-driven design.

## 5 Conclusions

In this study, we systematically evaluated multiple ligands’ pose generation strategies and their downstream impact on binding affinity prediction as part of the ASAP Antiviral Challenge 2025. Our results demonstrated that deep learning-based methods, exemplified by AlphaFold3, outperformed conventional methods in pose prediction, achieving the highest success rate and the lowest average RMSD for both SARS-CoV-2 and MERS-CoV Mpro targets. When integrated into our LRIP-SF machine learning workflow, AlphaFold3-based poses also yielded the most accurate potency predictions. AlphaFold3’s ability to generate biologically relevant and high-confidence poses highlighted its potential for guiding structure-based drug design, especially when no cocrystal data was available. Ligand superposition methods, exemplified by FlexS, while less accurate in pose prediction, offered a rapid and practical alternative that performs competitively in binding affinity prediction. In contrast, traditional docking methods such as Glide and AutoDock Vina, as well as Gnina which is built upon the AutoDock Vina engine, were computationally efficient but exhibited limited performance against the two main proteases.

To further interpret the mechanisms underlying binding affinity, we performed GSA on the LRIP-SF models. GSA identified key residues and residue groups that most significantly influenced binding affinity, such as H41, T135, and Q19–N119 in SARS-CoV-2 Mpro and C148, F143, and Q19 in MERS-CoV Mpro, many of which are conserved and located within the binding pocket. These insights offer valuable guidance to develop not only potent but also highly selective inhibitors.

Collectively, our findings underscore the pivotal role of accurate pose generation in enabling reliable machine learning-based affinity prediction and highlight the balance between computational efficiency and predictive power. The integration of deep learning-driven pose prediction with interaction-based scoring functions such as LRIP-SF demonstrates a powerful and generalizable framework for accelerating structure-based drug discovery.

## Supplementary Material



## Data Availability

All data used in this study was provided by the ASAP Discovery Consortium. These data sets are publicly available at the following URL: https://polarishub.io/organization/asap-discovery?artifactTypes=dataset&artifactTypes=benchmark. Molecular mechanics minimization and ligand–residue free energy decomposition were conducted with AMBER 24. Data analysis and machine learning model training were conducted with Python3. The source code for LRIP-SF has been deposited to zenodo.org (DOI: 10.5281/zenodo.16761312).

## Notes

Supporting Information includes detailed descriptions of the data sets used in the pose prediction and potency prediction subchallenges (Tables S1 and S2); performance comparisons of machine learning models on both protein targets (Tables S3–S16); Top-1/5/10 pose prediction success rates (Table S17); enrichment factor and hit-rate analyses (Tables S18–S21); 95% confidence intervals for potency-prediction evaluation metrics (Tables S22 and S23); global sensitivity analysis (GSA) results for both targets (Tables S24 and S25); hardware configurations and runtimes for each computational protocol (Table S26); paired *t*-test analyses for potency-prediction errors (Tables S27–S30); cross-docking analysis between MERS-CoV and SARS-CoV-2 Mpro (Figure S1); and correlation between AlphaFold3 ranking scores and experimentally determined binding affinities (Figure S2) (PDF)

## References

[1] Malone B., Urakova N., Snijder E. J., Campbell E. A. (2022). Structures and functions
of coronavirus replication-transcription complexes and their relevance
for SARS-CoV-2 drug design. Nat. Rev. Mol. Cell
Biol..

[2] Hu Q., Xiong Y., Zhu G. H., Zhang Y. N., Zhang Y. W., Huang P., Ge G. B. (2022). The SARS-CoV-2
main protease (M­(pro)):
Structure, function, and emerging therapies for COVID-19. MedComm.

[3] Jin Z., Du X., Xu Y., Deng Y., Liu M., Zhao Y., Zhang B., Li X., Zhang L., Peng C. (2020). Structure of M­(pro)
from SARS-CoV-2 and discovery of its inhibitors. Nature.

[4] Antiviral Drug Discovery 2025. https://polarishub.io/competitions/asap-discovery/antiviral-drug-discovery-2025 (accessed 2025).

[5] asap-discovery/antiviral-ligand-poses-2025. https://polarishub.io/competitions/asap-discovery/antiviral-ligand-poses-2025 (accessed 2025).

[6] asap-discovery/antiviral-potency-2025. https://polarishub.io/competitions/asap-discovery/antiviral-potency-2025 (accessed 2025).

[7] asap-discovery/antiviral-admet-2025. https://polarishub.io/competitions/asap-discovery/antiviral-admet-2025 (accessed 2025).

[8] Shen C., Hu X., Gao J., Zhang X., Zhong H., Wang Z., Xu L., Kang Y., Cao D., Hou T. (2021). The impact of cross-docked
poses on performance of machine learning classifier for protein-ligand
binding pose prediction. J. Cheminform..

[9] Friesner R. A. B. J.
L., Banks J. L., Murphy R. B., Halgren T. A., Klicic J. J., Mainz D. T., Repasky M. P., Knoll E. H., Shelley M., Perry J. K., Shaw D. E., Francis P. (2004). Glide:
A New Approach for Rapid, Accurate Docking and Scoring. 1. Method
and Assessment of Docking Accuracy. J. Med.
Chem..

[10] Trott O., Olson A. J. (2010). AutoDock Vina: improving
the speed and accuracy of
docking with a new scoring function, efficient optimization, and multithreading. J. Comput. Chem..

[11] Lemmen C., Lengauer T., Klebe G. (1998). FLEXS: A Method for Fast Flexible
Ligand Superposition. J. Med. Chem..

[12] Rarey M., Kramer B., Lengauer T., Klebe G. (1996). A fast flexible docking
method using an incremental construction algorithm. J. Mol. Biol..

[13] Abramson J., Adler J., Dunger J., Evans R., Green T., Pritzel A., Ronneberger O., Willmore L., Ballard A. J., Bambrick J. (2024). Accurate structure prediction of biomolecular
interactions with AlphaFold 3. Nature.

[14] Passaro S., Corso G., Wohlwend J., Reveiz M., Thaler S., Somnath V. R., Getz N., Portnoi T., Roy J., Stark H. (2025). Boltz-2:
Towards Accurate and Efficient Binding Affinity
Prediction. bioRxiv.

[15] Corso G., Stärk H., Jing B., Barzilay R., Jaakkola T. (2022). DiffDock:
Diffusion Steps, Twists, and Turns for Molecular Docking. arXiv.

[16] McNutt A. T., Li Y., Meli R., Aggarwal R., Koes D. R. (2025). GNINA 1.3: the next
increment in molecular docking with deep learning. J. Cheminform..

[17] Wohlwend J., Corso G., Passaro S., Getz N., Reveiz M., Leidal K., Swiderski W., Atkinson L., Portnoi T., Chinn I. (2025). Boltz-1
Democratizing Biomolecular Interaction Modeling. bioRxiv.

[18] Ragoza M., Hochuli J., Idrobo E., Sunseri J., Koes D. R. (2017). Protein-Ligand
Scoring with Convolutional Neural Networks. J. Chem. Inf. Model..

[19] Wang L., Wu Y., Deng Y., Kim B., Pierce L., Krilov G., Lupyan D., Robinson S., Dahlgren M. K., Greenwood J. (2015). Accurate and reliable
prediction of relative ligand binding potency
in prospective drug discovery by way of a modern free-energy calculation
protocol and force field. J. Am. Chem. Soc..

[20] He X., Liu S., Lee T. S., Ji B., Man V. H., York D. M., Wang J. (2020). Fast, Accurate, and
Reliable Protocols for Routine Calculations of
Protein-Ligand Binding Affinities in Drug Design Projects Using AMBER
GPU-TI with ff14SB/GAFF. ACS Omega.

[21] Wang E., Sun H., Wang J., Wang Z., Liu H., Zhang J. Z. H., Hou T. (2019). End-Point
Binding Free Energy Calculation with MM/PBSA
and MM/GBSA: Strategies and Applications in Drug Design. Chem. Rev..

[22] He X., Man V. H., Ji B., Xie X. Q., Wang J. (2019). Calculate
protein-ligand binding affinities with the extended linear interaction
energy method: application on the Cathepsin S set in the D3R Grand
Challenge 3. J. Comput. Aided Mol. Des..

[23] Onufriev A. V., Case D. A. (2019). Generalized Born
Implicit Solvent Models for Biomolecules. Annu.
Rev. Biophys..

[24] Hao D., He X., Ji B., Zhang S., Wang J. (2020). How Well Does the Extended
Linear Interaction Energy Method Perform in Accurate Binding Free
Energy Calculations?. J. Chem. Inf. Model..

[25] Ji B., He X., Zhai J., Zhang Y., Man V. H., Wang J. (2021). Machine learning
on ligand-residue interaction profiles to significantly improve binding
affinity prediction. Briefings Bioinf..

[26] Wang L., Ji B., Zhai J., Wang J. (2025). Advancing
promiscuous aggregating
inhibitor analysis with intelligent machine learning classification. Briefings Bioinf..

[27] Team, A. D. Ligand Poses (Blind Docking) – ASAP Polaris Blind Challenge Examples. 2025, https://github.com/asapdiscovery/asap-polaris-blind-challenge-examples/blob/main/02.%20Ligand%20Poses%20(Blind%20Docking).ipynb.

[28] O’Boyle N. M., Banck M., James C. A., Morley C., Vandermeersch T., Hutchison G. R. (2011). Open Babel:
An open chemical toolbox. J. Cheminform..

[29] Halgren T. A., Murphy R. B., Friesner R. A., Beard H. S., Frye L. L., Pollard W. T., Banks J. L. (2004). Glide: A New Approach for Rapid,
Accurate Docking and Scoring. 2. Enrichment Factors in Database Screening. J. Med. Chem..

[30] Schrödinger Suite 2017-1: Maestro, Version 11.2; Schrödinger, LLC: New York, NY, 2017.

[31] Madhavi
Sastry G., Adzhigirey M., Day T., Annabhimoju R., Sherman W. (2013). Protein and ligand preparation: parameters, protocols,
and influence on virtual screening enrichments. J. Comput. Aided Mol. Des..

[32] Harder E., Damm W., Maple J., Wu C., Reboul M., Xiang J. Y., Wang L., Lupyan D., Dahlgren M. K., Knight J. L. (2016). OPLS3: A Force Field
Providing Broad Coverage
of Drug-like Small Molecules and Proteins. J.
Chem. Theory Comput..

[33] Lu C., Wu C., Ghoreishi D., Chen W., Wang L., Damm W., Ross G. A., Dahlgren M. K., Russell E., Von Bargen C. D. (2021). OPLS4: Improving Force Field Accuracy on Challenging
Regimes of Chemical
Space. J. Chem. Theory Comput..

[34] Roos K., Wu C., Damm W., Reboul M., Stevenson J. M., Lu C., Dahlgren M. K., Mondal S., Chen W., Wang L. (2019). OPLS3e:
Extending Force Field Coverage for Drug-Like Small Molecules. J. Chem. Theory Comput..

[35] Johnston R. C., Yao K., Kaplan Z., Chelliah M., Leswing K., Seekins S., Watts S., Calkins D., Chief Elk J., Jerome S. V. (2023). Epik: pK­(a) and Protonation
State Prediction
through Machine Learning. J. Chem. Theory Comput..

[36] Morris G. M., Huey R., Lindstrom W., Sanner M. F., Belew R. K., Goodsell D. S., Olson A. J. (2009). AutoDock4
and AutoDockTools4: Automated
docking with selective receptor flexibility. J. Comput. Chem..

[37] Morris, G. M. ; Huey, R. ; Lindstrom, W. ; Sanner, M. F. ; Belew, R. K. ; Goodsell, D. S. ; Olson, A. J. AutoDockTools (ADT) Version 1.5.7. http://mgltools.scripps.edu/downloads (accessed 2025).

[38] Mirdita M., Schutze K., Moriwaki Y., Heo L., Ovchinnikov S., Steinegger M. (2022). ColabFold: making protein folding accessible to all. Nat. Methods.

[39] Niu T., Wang N., Wang J. (2024). Machine learning and deep learning
based scoring functions in deciphering ligand-receptor binding: An
application in drug design for GPCRs. Annu.
Rep. Comput. Chem..

[40] Hawkins G. D., Cramer C. J., Truhlar D. G. (1995). Pairwise
solute descreening of solute
charges from a dielectric medium. Chem. Phys.
Lett..

[41] Hawkins G. D., Cramer C. J., Truhlar D. G. (1996). Parametrized
Models of Aqueous Free
Energies of Solvation Based on Pairwise Descreening of Solute Atomic
Charges from a Dielectric Medium. J. Phys. Chem..

[42] Maier J. A., Martinez C., Kasavajhala K., Wickstrom L., Hauser K. E., Simmerling C. (2015). ff14SB: Improving the Accuracy of
Protein Side Chain and Backbone Parameters from ff99SB. J. Chem. Theory Comput..

[43] Wang J. M., Wolf R. M., Caldwell J. W., Kollman P. A., Case D. A. (2004). Development
and testing of a general amber force field. J. Comput. Chem..

[44] He X., Man V. H., Yang W., Lee T. S., Wang J. (2020). A fast and
high-quality charge model for the next generation general AMBER force
field. J. Chem. Phys..

[45] He X., Man V. H., Yang W., Lee T. S., Wang J. (2025). ABCG2: A Milestone
Charge Model for Accurate Solvation Free Energy Calculation. J. Chem. Theory Comput..

[46] Case, D. A. ; Aktulga, H. M. ; Belfon, K. ; Ben-Shalom, I. Y. ; Berryman, J. T. ; Brozell, S. R. ; Carvahol, F. S. ; Cerutti, D. S. ; Cheatham, T. E., III ; Cisneros, G. A. ; Cruzeiro, V. W. D. ; Darden, T. A. ; Forouzesh, N. ; Ghazimirsaeed, M. ; Giambaşu, G. ; Giese, T. ; Gilson, M. K. ; Gohlke, H. ; Goetz, A. W. ; Harris, J. ; Huang, Z. ; Izadi, S. ; Izmailov, S. A. ; Kasavajhala, K. ; Kaymak, M. C. ; Kolossv’a ry, I. ; Kovalenko, A. ; Kurtzman, T. ; Lee, T. S. ; Li, P. ; Li, Z. ; Lin, C. ; Liu, J. ; Luchko, T. ; Luo, R. ; Machado, M. ; Manathunga, M. ; Merz, K. M. ; Miao, Y. ; Mikhailovskii, O. ; Monard, G. ; Nguyen, H. ; O’Hearn, K. A. ; Onufriev, A. ; Pan, F. ; Pantano, S. ; Rahnamoun, A. ; Roe, D. R. ; Roitberg, A. ; Sagui, C. ; Schott-Verdugo, S. ; Shajan, A. ; Shen, J. ; Simmerling, C. L. ; Skrynnikov, N. R. ; Smith, J. ; Swails, J. ; Walker, R. C. ; Wang, J. ; Wang, J. ; Wu, X. ; Wu, Y. ; Xiong, Y. ; Xue, Y. ; York, D. M. ; Zhao, C. ; Zhu, Q. ; Kollman, P. A. Amber 2025; University of California: San Francisco, 2025.

[47] Case D. A., Aktulga H. M., Belfon K., Cerutti D. S., Cisneros G. A., Cruzeiro V. W. D., Forouzesh N., Giese T. J., Gotz A. W., Gohlke H. (2023). AmberTools. J. Chem. Inf. Model..

[48] Wang J., Wang W., Kollman P. A., Case D. A. (2006). Automatic
atom type
and bond type perception in molecular mechanical calculations. J. Mol. Graph. Model..

[49] Report on protein–ligand pose prediction for viral proteases in the ASAP 2025 competition. 2025, https://docs.google.com/document/d/1SGw3gklEnCv-n_zV3mRvpl4sok304032/edit?tab=t.0#heading=h.gjdgxs (accessed 2025).

[50] Antiviral Drug Discovery 2025 Results. https://polarishub.io/competitions/asap-discovery/antiviral-drug-discovery-2025#competiton-results (accessed 2025).

[51] Vithayapalert, W. Ligand Pose Prediction by a 2-Stage Structure Prediction Framework with Fine-Tuned Boltz-1 and DiffDock-L. 2025, https://apricot-zorah-51.tiiny.site/ (accessed 2025).

[52] Mendez-Lucio O., Nicolaou C. A., Earnshaw B. (2024). MolE: a foundation model for molecular
graphs using disentangled attention. Nat. Commun..

[53] Overview for submission for Polaris Antiviral Competition 2025. https://gist.github.com/shug3502/f0fe74f443d1d82fa73bf869148c7cdc (accessed 2025).

[54] Predicted pIC50 for the two protein targets. https://drive.google.com/file/d/1axeFC3gxcVGKPDwVZO2Tyi7W9Mz2ztLN/view (accessed 2025).

[55] Kalliokoski T., Kramer C., Vulpetti A., Gedeck P. (2013). Comparability of mixed
IC(5)(0) data - a statistical analysis. PLoS
One.

[56] Kramer C., Kalliokoski T., Gedeck P., Vulpetti A. (2012). The experimental uncertainty
of heterogeneous public K­(i) data. J. Med. Chem..

