# Supplementary material for: A Comparative Study of Deep Learning and Classical Modeling Approaches for Protein–Ligand Binding Pose and Affinity Prediction in Coronavirus Main Proteases
Source: J Chem Inf Model. 2025 Dec 22;66(1):731–43. doi: 10.1021/acs.jcim.5c02481 (PMC12801289; doi:10.1021/acs.jcim.5c02481)
Supplement: Supplementary file 1 [file ci5c02481_si_001.pdf]

Supporting Information

A Comparative Study of Deep Learning and Classical Modeling Approaches for Protein–Ligand Binding Pose and Affinity Prediction in Coronavirus Main Proteases

Yue Liu[1], Haocheng Tang[1], Taoyu Niu[1], Junmei Wang[1*]

[1]Department of Pharmaceutical Sciences and Computational Chemical Genomics Screening Center, School of Pharmacy, University of Pittsburgh, Pittsburgh, Pennsylvania 15261, United States

*: corresponding author:

Junmei Wang juw79@pitt.edu

Contents

Table S1. Data structure of the binding pose prediction dataset.

Table S2. Data structure of the potency prediction dataset.

Table S3–S16. Performance of machine learning models in LRIP-SF affinity prediction using poses generated by Glide, AutoDock Vina, FlexS, AlphaFold3, Boltz-2, DiffDock, and Gnina (Rigid Receptor) for both MERS-CoV and SARS-CoV-2 Mpro.

Table S17. Top-1, Top-5, and Top-10 pose prediction success rates across all pose-generation methods.

Table S18–S21. Enrichment factors and hit rates at multiple cutoffs (1–40%) for MERS-CoV and SARS-CoV-2 Mpro.

Table S22–S23. 95% confidence intervals for potency-prediction metrics for all methods on both targets.

Table S24–S25. Global Sensitivity Analysis (GSA) results for MERS-CoV and SARS-CoV-2 Mpro.

Table S26. Hardware configurations and computational runtimes for each method.

Table S27–S30. Statistical significance analysis of MAE and RMSE differences between AlphaFold3 and other methods.

Figure S1. Cross-docking analysis between MERS-CoV and SARS-CoV-2 Mpro.

Figure S2. Correlation between AlphaFold3 ranking score and binding affinity for each Mpro target

Table S1. Data structure of the dataset that was used for the binding pose prediction sub-challenge. Each entry includes sequence, structural, and ligand information. Reference protein structures for SARS-CoV-2 and MERS-CoV Mpro were provided by the challenge for pose alignment and are available at: https://fs.polarishub.io/2025-01-asap-discovery/ligand_poses_reference_structures.zip.

Column	Data Type	Description
Chain A Sequence	Str	Primary structure of the protein's A chain: A linear sequence of amino acids.
Chain B Sequence	Str	Primary structure of the protein's B chain, if any: A linear sequence of amino acids.
CXSMILES	str	Text representation of the 2D molecular structure.
Complex Structure	PDB	3D system of the ligand bound to the protein, prepared using OESpruce and aligned to a reference Mpro.
Protein Structure	PDB	3D system of just the protein structure, prepared using OESpruce and aligned to a reference Mpro.
Ligand Pose	SDF	3D conformation of the molecule, bound to the protein.
Protein Label	str	Either SARS-CoV-2 Mpro or MERS-CoV Mpro.

Table S2. Data structure of the dataset that was used for the potency prediction sub-challenge. Each entry includes the molecule's identifier, 2D structure in CXSMILES format, and experimentally measured pIC_{50} values against SARS-CoV-2 Mpro and MERS-CoV Mpro.

Column	Data Type	Description
Molecule Name	Str	Internal identifier at ASAP Discovery for this molecule.

CXSMILES	str	Text representation of the 2D molecular structure.
pIC_{50} (SARS-CoV-2 Mpro)	float	Negative log10 of the IC_{50} values of the dose-response curve.
pIC_{50} (MERS-CoV Mpro)	float	Negative log10 of the IC_{50} values of the dose-response curve.

Table S3. Performance of machine learning models in LRIP-SF binding affinity prediction for MERS-CoV Mpro using poses predicted by Glide. Model evaluation was based on six metrics: Root Mean Squared Error (RMSE), Mean Absolute Error (MAE), Pearson Correlation Coefficient (Pearson R), Coefficient of Determination ($CORR^2$), Prediction Index (PI), Kendall's Tau (TAU) and p-value. GBDT demonstrated the strongest predictive performance across all evaluation criteria.

ML Algorithm	RMSE	MAE	Pearson's R	CORR2	PI	TAU	p-value
LR	0.753	0.500	0.434	0.188	0.472	0.296	1.965e-39
LASSO	0.801	0.565	0.314	0.099	0.352	0.207	1.848e-20
Bayesian	0.774	0.528	0.378	0.143	0.415	0.252	1.179e-29
SVR	0.753	0.441	0.466	0.217	0.509	0.373	5.240e-46
RF	0.608	0.458	0.757	0.574	0.645	0.349	2.187e-155
Adaboost	0.697	0.604	0.707	0.500	0.558	0.295	6.256e-127
GBDT	0.410	0.316	0.904	0.818	0.852	0.574	3.310e-308
MLP	0.475	0.317	0.832	0.692	0.817	0.555	2.372e-214

Table S4. Performance of machine learning models for the SARS-CoV-2 Mpro target in the LRIP-SF framework using poses predicted by Glide. Model evaluation was based on

six metrics: Root Mean Squared Error (RMSE), Mean Absolute Error (MAE), Pearson Correlation Coefficient (Pearson R), Coefficient of Determination ($CORR^2$), Prediction Index (PI), Kendall's Tau (TAU) and p-value. GBDT demonstrated the strongest predictive performance across all evaluation criteria.

ML Algorithm	RMSE	MAE	Pearson's R	CORR2	PI	TAU	p-value
LR	0.768	0.609	0.643	0.413	0.601	0.373	3.298e-98
LASSO	0.901	0.730	0.463	0.214	0.474	0.276	2.305e-45
Bayesian	0.827	0.666	0.566	0.320	0.528	0.314	1.550e-71
SVR	0.764	0.578	0.650	0.422	0.612	0.394	5.336e-101
RF	0.628	0.518	0.813	0.661	0.747	0.497	5.485e-197
Adaboost	0.754	0.653	0.755	0.570	0.691	0.447	1.779e-154
GBDT	0.468	0.377	0.898	0.807	0.881	0.668	2.436e-298
MLP	0.593	0.457	0.843	0.711	0.813	0.584	7.712e-226

Table S5. Performance of machine learning models for the MERS-CoV Mpro target in the LRIP-SF framework using poses predicted by AutoDock Vina. Model evaluation was based on six metrics: Root Mean Squared Error (RMSE), Mean Absolute Error (MAE), Pearson Correlation Coefficient (Pearson R), Coefficient of Determination ($CORR^2$), Prediction Index (PI), Kendall's Tau (TAU) and p-value. GBDT demonstrated the strongest predictive performance across all evaluation criteria.

ML Algorithm	RMSE	MAE	Pearson's R	CORR2	PI	TAU	p-value
LR	0.723	0.475	0.501	0.251	0.534	0.332	2.823e-53
LASSO	0.810	0.574	0.277	0.059	0.306	0.193	6.457e-16
Bayesian	0.784	0.535	0.351	0.119	0.368	0.237	3.7747e-25
SVR	0.773	0.457	0.428	0.144	0.461	0.347	7.265e-38
RF	0.580	0.434	0.783	0.517	0.679	0.380	2.940e-171
Adaboost	0.636	0.557	0.755	0.420	0.584	0.316	1.771e-152
GBDT	0.403	0.306	0.908	0.768	0.864	0.603	2.242e-311

MLP	0.751	0.310	0.866	0.708	0.849	0.588	3.086e-249

Table S6. Performance of machine learning models for the SARS-CoV-2 Mpro target in the LRIP-SF framework using poses predicted by AutoDock Vina. Model evaluation was based on six metrics: Root Mean Squared Error (RMSE), Mean Absolute Error (MAE), Pearson Correlation Coefficient (Pearson R), Coefficient of Determination ($CORR^2$), Prediction Index (PI), Kendall's Tau (TAU), and p-value. GBDT demonstrated the strongest predictive performance across all evaluation criteria.

ML Algorithm	RMSE	MAE	Pearson's R	CORR2	PI	TAU	p-value
LR	0.711	0.551	0.707	0.500	0.671	0.434	3.173e-125
LASSO	0.947	0.771	0.351	0.112	0.349	0.212	3.198e-25
Bayesian	0.802	0.644	0.606	0.364	0.577	0.354	3.360e-83
SVR	0.774	0.595	0.644	0.407	0.613	0.395	2.098e-97
RF	0.624	0.510	0.807	0.614	0.762	0.519	1.756e-189
Adaboost	0.690	0.593	0.788	0.529	0.712	0.469	1.046e-174
GBDT	0.463	0.372	0.903	0.788	0.891	0.687	1.719e-302
MLP	0.462	0.345	0.892	0.788	0.872	0.666	8.162e-284

Table S7. Performance of machine learning models for the MERS-CoV Mpro target in the LRIP-SF framework using poses predicted by FlexS. Model evaluation was based on six metrics: Root Mean Squared Error (RMSE), Mean Absolute Error (MAE), Pearson Correlation Coefficient (Pearson R), Coefficient of Determination ($CORR^2$),

Prediction Index (PI), Kendall’s Tau (TAU) and p-value. GBDT demonstrated the strongest predictive performance across all evaluation criteria.

ML Algorithm	RMSE	MAE	Pearson’s R	CORR2	PI	TAU	p-value
LR	0.680	0.444	0.512	0.262	0.540	0.323	2.516e-52
LASSO	0.782	0.558	0.173	0.025	0.210	0.103	1.483e-6
Bayesian	0.790	0.566	0.084	0.006	0.134	0.035	0.020
SVR	0.831	0.536	0.073	-0.101	-0.055	-0.014	0.043
RF	0.515	0.386	0.816	0.577	0.735	0.441	1.223e-183
Adaboost	0.542	0.470	0.778	0.531	0.545	0.266	1.160e-155
GBDT	0.350	0.263	0.925	0.805	0.883	0.625	2e-323
MLP	3.721	0.632	0.064	-21.060	0.392	0.227	0.078

Table S8. Performance of machine learning models for the SARS-CoV-2 Mpro target in the LRIP-SF framework using poses predicted by FlexS. Model evaluation was based on six metrics: Root Mean Squared Error (RMSE), Mean Absolute Error (MAE), Pearson Correlation Coefficient (Pearson R), Coefficient of Determination ($CORR^2$), Prediction Index (PI), Kendall’s Tau (TAU) and p-value. GBDT demonstrated the strongest predictive performance across all evaluation criteria.

ML Algorithm	RMSE	MAE	Pearson’s R	CORR2	PI	TAU	p-value
LR	0.709	0.574	0.678	0.459	0.598	0.354	5.398e-105
LASSO	0.930	0.745	0.288	0.071	0.290	0.160	2.894e-16
Bayesian	0.765	0.625	0.612	0.372	0.547	0.313	1.743e-80
SVR	0.975	0.744	0.136	-0.021	-0.015	0.007	0.000
RF	0.579	0.472	0.824	0.640	0.783	0.546	2.531e-192
Adaboost	0.677	0.584	0.786	0.508	0.728	0.490	2.348e-163
GBDT	0.387	0.309	0.927	0.839	0.916	0.723	0.000
MLP	7.414	0.913	-0.039	-58.067	0.503	0.288	0.284

Table S9. Performance of machine learning models for the MERS-CoV Mpro target in the LRIP-SF framework using poses predicted by AlphaFold3. Model evaluation was based on six metrics: Root Mean Squared Error (RMSE), Mean Absolute Error (MAE), Pearson Correlation Coefficient (Pearson R), Coefficient of Determination ($CORR^2$), Prediction Index (PI), Kendall's Tau (TAU) and p-value. GBDT demonstrated the strongest predictive performance across all evaluation criteria.

ML Algorithm	RMSE	MAE	Pearson's R	CORR2	PI	TAU	p-value
LR	0.707	0.469	0.532	0.283	0.560	0.353	5.223e-61
LASSO	0.826	0.591	0.209	0.021	0.316	0.226	1.592e-9
Bayesian	0.766	0.512	0.402	0.157	0.446	0.308	3.203e-33
SVR	0.769	0.452	0.426	0.152	0.465	0.357	1.548e-37
RF	0.571	0.416	0.788	0.532	0.727	0.435	2.697e-174
Adaboost	0.611	0.526	0.749	0.463	0.537	0.258	3.334e-148
GBDT	0.368	0.278	0.922	0.805	0.887	0.632	0.000
MLP	0.634	0.416	0.659	0.422	0.681	0.445	3.068e-103

Table S10. Performance of machine learning models for the SARS-CoV-2 Mpro target in the LRIP-SF framework using poses predicted by AlphaFold3. Model evaluation was based on six metrics: Root Mean Squared Error (RMSE), Mean Absolute Error (MAE), Pearson Correlation Coefficient (Pearson R), Coefficient of Determination ($CORR^2$), Prediction Index (PI), Kendall's Tau (TAU) and p-value. GBDT demonstrated the strongest predictive performance across all evaluation criteria.

ML Algorithm	RMSE	MAE	Pearson's R	CORR2	PI	TAU	p-value
LR	0.644	0.493	0.767	0.589	0.757	0.531	1.585e-159
LASSO	0.845	0.688	0.581	0.291	0.578	0.357	5.125e-75

Bayesian	0.725	0.573	0.692	0.478	0.657	0.424	1.555e-117
SVR	0.995	0.764	0.322	0.018	0.564	0.352	3.197e-21
RF	0.519	0.411	0.867	0.733	0.846	0.624	3.813e-249
Adaboost	0.633	0.550	0.837	0.700	0.802	0.573	2.287e-215
GBDT	0.376	0.289	0.933	0.860	0.925	0.749	0.000
MLP	0.779	0.618	0.637	0.399	0.623	0.391	2.314e-94

Table S11. Performance of machine learning models for the MERS-CoV Mpro target in the LRIP-SF framework using poses predicted by Boltz-2. Model evaluation was based on six metrics: Root Mean Squared Error (RMSE), Mean Absolute Error (MAE), Pearson Correlation Coefficient (Pearson R), Coefficient of Determination ($CORR^2$), Prediction Index (PI), Kendall's Tau (TAU) and p-value. GBDT demonstrated the strongest predictive performance across all evaluation criteria.

ML Algorithm	RMSE	MAE	CORR	CORR2	p-value	PI	TAU
LR	0.747	0.500	0.445	0.198	1.634E-41	0.485	0.299
LASSO	0.829	0.598	0.163	0.011	2.486E-06	0.254	0.138
Bayesian	0.781	0.532	0.359	0.124	1.492E-26	0.376	0.260
SVR	0.804	0.499	0.338	0.070	1.377E-23	0.368	0.288
RF	0.585	0.430	0.771	0.508	1.650E-164	0.678	0.390
Adaboost	0.603	0.519	0.753	0.477	1.008E-152	0.523	0.261
GBDT	0.387	0.288	0.913	0.784	1e-323	0.878	0.626
MLP	0.955	0.453	0.395	-0.310	2.492E-32	0.690	0.437

Table S12. Performance of machine learning models for the SARS-CoV-2 Mpro target in the LRIP-SF framework using poses predicted by Boltz-2. Model evaluation was based on six metrics: Root Mean Squared Error (RMSE), Mean Absolute Error (MAE), Pearson Correlation Coefficient (Pearson R), Coefficient of Determination ($CORR^2$), Prediction Index (PI), Kendall's Tau (TAU) and p-value. GBDT demonstrated the strongest predictive performance across all evaluation criteria.

ML Algorithm	RMSE	MAE	CORR	CORR2	p-value	PI	TAU
LR	0.672	0.514	0.746	0.556	4.991E-148	0.727	0.499
LASSO	0.830	0.667	0.595	0.322	2.148E-80	0.570	0.369
Bayesian	0.715	0.554	0.706	0.498	5.318E-126	0.684	0.463
SVR	0.900	0.699	0.587	0.203	4.530E-78	0.592	0.387
RF	0.537	0.421	0.854	0.717	3.755E-237	0.850	0.629
Adaboost	0.615	0.523	0.835	0.628	1.943E-216	0.805	0.575
GBDT	0.382	0.294	0.932	0.857	0	0.925	0.745
MLP	1.145	0.607	0.460	-0.290	1.083E-44	0.682	0.452

Table S13. Performance of machine learning models for the MERS-CoV Mpro target in the LRIP-SF framework using poses predicted by DiffDock. Model evaluation was based on six metrics: Root Mean Squared Error (RMSE), Mean Absolute Error (MAE), Pearson Correlation Coefficient (Pearson R), Coefficient of Determination ($CORR^2$), Prediction Index (PI), Kendall's Tau (TAU) and p-value. GBDT demonstrated the strongest predictive performance across all evaluation criteria.

ML Algorithm	RMSE	MAE	CORR	CORR2	p-value	PI	TAU
LR	0.739	0.495	0.464	0.216	9.523E-46	0.513	0.303
LASSO	0.824	0.591	0.229	0.026	2.260E-11	0.313	0.194
Bayesian	0.774	0.532	0.381	0.141	4.407E-30	0.438	0.255
SVR	0.830	0.527	0.326	0.010	4.349E-22	0.382	0.261
RF	0.587	0.446	0.783	0.505	1.197E-173	0.687	0.396
Adaboost	0.659	0.584	0.732	0.377	2.492E-140	0.470	0.220
GBDT	0.383	0.296	0.917	0.789	0	0.872	0.608
MLP	1.111	0.530	0.252	-0.773	1.679E-13	0.539	0.309

Table S14. Performance of machine learning models for the SARS-CoV-2 Mpro target in the LRIP-SF framework using poses predicted by DiffDock. Model evaluation was based on six metrics: Root Mean Squared Error (RMSE), Mean Absolute Error (MAE),

Pearson Correlation Coefficient (Pearson R), Coefficient of Determination (CORR²), Prediction Index (PI), Kendall's Tau (TAU) and p-value. GBDT demonstrated the strongest predictive performance across all evaluation criteria.

ML Algorithm	RMSE	MAE	CORR	CORR2	p-value	PI	TAU
LR	0.782	0.626	0.628	0.395	2.58E-92	0.585	0.348
LASSO	0.916	0.754	0.438	0.170	3.11E-40	0.423	0.222
Bayesian	0.848	0.683	0.539	0.289	9.32E-64	0.500	0.282
SVR	1.030	0.790	0.039	-0.049	0.257519	0.104	0.067
RF	0.632	0.513	0.806	0.605	8.9E-191	0.755	0.507
Adaboost	0.702	0.607	0.775	0.512	4.8E-167	0.717	0.476
GBDT	0.442	0.354	0.916	0.807	0	0.907	0.706
MLP	1.083	0.747	0.408	-0.160	1.41E-34	0.495	0.288

Table S15. Performance of machine learning models for the MERS-CoV Mpro target in the LRIP-SF framework using poses predicted by Gnina (Rigid Receptor). Model evaluation was based on six metrics: Root Mean Squared Error (RMSE), Mean Absolute Error (MAE), Pearson Correlation Coefficient (Pearson R), Coefficient of Determination (CORR²), Prediction Index (PI), Kendall's Tau (TAU) and p-value. GBDT demonstrated the strongest predictive performance across all evaluation criteria.

ML Algorithm	RMSE	MAE	CORR	CORR2	p-value	PI	TAU
LR	0.745	0.506	0.453	0.205	3.393E-43	0.456	0.296
LASSO	0.809	0.571	0.265	0.063	7.946E-15	0.315	0.200
Bayesian	0.770	0.525	0.392	0.151	7.717E-32	0.410	0.268
SVR	0.758	0.453	0.458	0.176	2.316E-44	0.462	0.329
RF	0.605	0.452	0.747	0.475	3.864E-149	0.656	0.367
Adaboost	0.663	0.562	0.732	0.371	2.746E-140	0.588	0.331
GBDT	0.429	0.322	0.893	0.736	6.486E-289	0.849	0.582
MLP	0.674	0.501	0.667	0.348	7.993E-108	0.667	0.426

Table S16. Performance of machine learning models for the SARS-CoV-2 Mpro target in the LRIP-SF framework using poses predicted by Gnina (Rigid Receptor). Model evaluation was based on six metrics: Root Mean Squared Error (RMSE), Mean Absolute Error (MAE), Pearson Correlation Coefficient (Pearson R), Coefficient of Determination (CORR²), Prediction Index (PI), Kendall's Tau (TAU) and p-value. GBDT demonstrated the strongest predictive performance across all evaluation criteria.

ML Algorithm	RMSE	MAE	CORR	CORR2	p-value	PI	TAU
LR	0.776	0.627	0.637	0.406	3.753E-96	0.594	0.358
LASSO	0.905	0.738	0.454	0.192	1.435E-43	0.430	0.244
Bayesian	0.834	0.676	0.562	0.315	1.137E-70	0.534	0.311
SVR	0.776	0.597	0.641	0.406	1.808E-97	0.600	0.375
RF	0.647	0.529	0.805	0.588	1.717E-190	0.766	0.520
Adaboost	0.727	0.632	0.759	0.479	4.084E-157	0.684	0.440
GBDT	0.484	0.391	0.898	0.769	5.400E-299	0.877	0.662
MLP	0.411	0.304	0.915	0.833	0	0.893	0.692

Table S17 Top1, Top 5 and Top 10 Pose Prediction Success rate

Method	Total	Top_1	Top_5	Top_10
AlphaFold3	194	0.876	0.912	0.912
Boltz-2	195	0.841	0.877	0.882
FlexS	194	0.459	0.67	0.711
DiffDock	195	0.123	0.205	0.231
Glide	195	0.246	0.538	0.569
Gnina-RR	195	0.021	0.067	0.097

AutoDock Vina	195	0.031	0.077	0.087

Table S18 EF at 1%, 5%, 10%, 20%, 40% for predictions against MERS-CoV Mpro. Ligands with experimentally determined pIC_{50} > 6 are considered as actives.

Method	Total Hits	EF_1%	EF_5%	EF_10%	EF_20%	EF_40%
AlphaFold3	76	3.842	3.019	3.047	2.782	2.219
Glide	76	0	1.921	1.987	1.855	1.888
FlexS	72	3.958	2.827	2.686	2.569	2.188
Gnina-scoring	76	3.842	2.744	2.517	1.921	1.59
DiffDock	75	1.94	2.771	2.676	2.208	1.94
Boltz-2	75	1.94	2.771	3.077	2.609	1.973
Boltz2-Internal	75	1.94	3.326	2.81	2.542	2.308
Gnina-RR	76	1.901	2.445	2.445	2.202	1.984
AutoDock Vina	76	3.842	2.744	2.517	2.385	2.252

Table S19 Hit rate at 1%, 5%, 10%, 20%, 40% for MERS-CoV Mpro. Ligands with experimentally determined pIC_{50} > 6 are considered as actives.

Method	Total Hits	Top_1% Hit Rate	Top_5% Hit Rate	Top_10% Hit Rate	Top_20% Hit Rate	Top_40% Hit Rate

AlphaFold3	76	1	0.786	0.793	0.724	0.578
Glide	76	0	0.5	0.517	0.483	0.491
FlexS	72	1	0.714	0.679	0.649	0.553
Gnina-scoring	76	1	0.714	0.655	0.5	0.414
DiffDock	75	0.5	0.714	0.69	0.569	0.5
Boltz-2	75	0.5	0.714	0.793	0.672	0.509
Boltz2-internal	75	0.5	0.857	0.724	0.655	0.595
Gnina-RR	76	0.5	0.643	0.643	0.579	0.522
AutoDock Vina	76	1	0.714	0.655	0.621	0.586

Table S20 EF at 1%, 5%, 10%, 20%, 40% for predictions against SARS-CoV-2 Mpro. Ligands with experimentally determined pIC_{50} > 6 are considered as actives.

Method	Total Hits	EF_1%	EF_5%	EF_10%	EF_20%	EF_40%
AlphaFold3	146	1.767	1.767	1.626	1.663	1.647
Glide	146	1.767	1.767	1.767	1.767	1.664
FlexS	139	1.799	1.799	1.799	1.799	1.673
Gnina-Scoring	146	1.767	1.767	1.626	1.594	1.596
DiffDock	145	1.772	1.625	1.702	1.703	1.651
Boltz-2	145	1.772	1.772	1.772	1.703	1.633
Boltz2-Internal	145	1.772	1.772	1.772	1.772	1.738
Gnina-RR	146	1.753	1.607	1.613	1.65	1.564

AutoDock Vina	146	1.767	1.767	1.555	1.629	1.664

Table S21 Hit rate at 1%, 5%, 10%, 20%, 40% for predictions against SARS-CoV-2 Mpro. Ligands with experimentally determined pIC_{50} > 6 are considered as actives.

Method	Total Hits	Top_1% Hit Rate	Top_5% Hit Rate	Top_10 % Hit Rate	Top_20 % Hit Rate	Top_40 % Hit Rate
AlphaFold3	146	1	1	0.92	0.941	0.932
Glide	146	1	1	1	1	0.942
FlexS	139	1	1	1	1	0.93
Gnina-Scoring	146	1	1	0.92	0.902	0.903
DiffDock	145	1	0.917	0.96	0.961	0.931
Boltz-2	145	1	1	1	0.961	0.922
Boltz2-Internal	145	1	1	1	1	0.98
Gnina-RR	146	1	0.917	0.92	0.941	0.892
AutoDock Vina	146	1	1	0.88	0.922	0.942

Table S22 95% Confidence Interval for potency prediction evaluation metrics for MERS-CoV Mpro.

Method	MAE	RMSE	Pearson's R	R^2	Kendall's tau	PI
AlphaFold3	0.606[0.547,0.676]	0.813[0.704,0.940]	0.643[0.542,0.734]	0.340[0.222,0.458]	0.477[0.414,0.529]	0.739[0.708,0.765]
Boltz-2-Internal	1.470[1.385,1.567]	1.672[1.573,1.785]	0.696[0.570,0.799]	-1.805[-2.320, -1.388]	0.556[0.502,0.606]	0.779[0.751,0.804]
Boltz-2	0.658[0.592,0.728]	0.896[0.780,1.038]	0.538[0.379,0.675]	0.197[0.028,0.342]	0.447[0.381,0.513]	0.724[0.691,0.757]
DiffDock	0.676[0.614,0.746]	0.898[0.797,1.030]	0.575[0.459,0.667]	0.194[0.063,0.306]	0.446[0.388,0.500]	0.723[0.694,0.751]
FlexS	0.612[0.553,0.682]	0.828[0.713,0.960]	0.626[0.519,0.718]	0.309[0.165,0.436]	0.493[0.439,0.546]	0.747[0.720,0.774]
Glide	0.737[0.667,0.811]	0.961[0.861,1.079]	0.442[0.327,0.549]	0.078[-0.038,0.189]	0.354[0.294,0.412]	0.677[0.647,0.706]
Gnina-RR	0.712[0.646,0.784]	0.929[0.828,1.032]	0.491[0.390,0.583]	0.135[0.027,0.239]	0.380[0.324,0.437]	0.690[0.662,0.719]
Gnina-Scoring	2.178[1.965,2.402]	2.834[2.547,3.126]	0.216[0.072,0.343]	-7.049[-8.660, -5.588]	0.220[0.137,0.299]	0.610[0.569,0.650]
AutoDock Vina	0.681[0.623,0.745]	0.873[0.782,0.978]	0.588[0.493,0.678]	0.239[0.131,0.340]	0.475[0.419,0.526]	0.738[0.710,0.763]

Table S23 95% Confidence Interval for potency prediction evaluation metrics for SARS-CoV-2 Mpro.

Method	MAE	RMSE	Pearson's R	R2	Kendall's tau	PI
AlphaFold3	0.724[0.660,0.789]	0.894[0.823,0.973]	0.820[0.765,0.868]	0.548[0.473,0.618]	0.607[0.554,0.656]	0.804[0.778,0.828]
Boltz-2-Internal	0.950[0.874,1.024]	1.133[1.035,1.232]	0.845[0.803,0.884]	0.272[0.130,0.403]	0.638[0.595,0.683]	0.819[0.798,0.842]
Boltz-2	0.716[0.648,0.790]	0.909[0.831,1.000]	0.800[0.740,0.849]	0.532[0.442,0.607]	0.598[0.546,0.642]	0.799[0.773,0.822]
DiffDock	0.973[0.888,1.060]	1.192[1.106,1.280]	0.695[0.614,0.759]	0.195[0.075,0.309]	0.512[0.456,0.565]	0.756[0.728,0.783]
FlexS	0.722[0.653,0.797]	0.925[0.838,1.015]	0.782[0.736,0.826]	0.510[0.417,0.595]	0.587[0.539,0.632]	0.794[0.770,0.816]
Glide	0.860[0.780,0.944]	1.070[0.977,1.162]	0.724[0.664,0.774]	0.352[0.238,0.455]	0.524[0.470,0.573]	0.762[0.735,0.787]
Gnina-RR	1.000[0.912,1.104]	1.253[1.154,1.363]	0.638[0.559,0.706]	0.101[-0.052,0.234]	0.450[0.391,0.505]	0.725[0.696,0.753]
Gnina-Scoring	4.190[3.568,4.917]	6.871[5.779,8.109]	0.350[0.264,0.429]	-25.954[-36.919, s-17.258]	0.414[0.351,0.475]	0.707[0.676,0.738]
AutoDock Vina	0.862[0.781,0.939]	1.067[0.978,1.148]	0.718[0.655,0.775]	0.355[0.248,0.457]	0.531[0.478,0.581]	0.766[0.739,0.790]

Table S24. Results of Global Sensitivity Analysis (GSA) for the MERS-CoV Mpro target. Residue groups with a Pearson correlation coefficient greater than 0.85 are ranked by their impact on model performance, as measured by Root Mean Squared Error (RMSE). Higher RMSE values indicate greater importance. The baseline RMSE, representing the model without perturbation, is provided for reference.

Rank	RMSE	Residue Group
1	0.7560	148C
2	0.7517	39P, 177G
3	0.7509	143F
4	0.7496	19Q
5	0.7490	52P
6	0.7488	252D, 262I, 263E
7	0.7488	196V
8	0.7486	261A
9	0.7486	25M
10	0.7480	225P
11	0.7477	226N,260V
12	0.7474	26T
13	0.7472	41H
14	0.7464	149G
15	0.7463	122N
16	0.7462	192Q
17	0.7461	Baseline

Table S25. Results of Global Sensitivity Analysis (GSA) for the SARS-CoV-2 Mpro target. Residue groups with a Pearson correlation coefficient greater than 0.85 are ranked by their impact on model performance, as measured by Root Mean Squared

Error (RMSE). Higher RMSE values indicate greater importance. The baseline RMSE, representing the model without perturbation, is provided for reference.

Rank	RMSE	Residue Group	Rank	RMSE	Residue Group
1	0.8043	41H	26	0.7840	145C
2	0.7937	135T	27	0.7838	181F
3	0.7931	19Q, 119N	28	0.7836	118Y
4	0.7928	191A	29	0.7835	54Y
5	0.7918	43I, 44C, 45T, 48D, 49M, 164H	30	0.7833	129A, 133N, 237Y
6	0.7908	138G, 139S, 172H	31	0.7827	27L
7	0.7902	23G	32	0.7826	116A
8	0.7897	55E, 270E, 273Q	33	0.7826	185F
9	0.7869	167L	34	0.7826	143G
10	0.7866	40R	35	0.7824	193A, 194A
11	0.7862	173A	36	0.7823	141L
12	0.7862	170G	37	0.7822	115L
13	0.7854	25T	38	0.7822	50, 57, 83, 179G
14	0.7853	168P	39	0.7822	52P
15	0.7853	46S	40	0.7821	22C
16	0.7852	117C	41	0.7818	186V
17	0.7851	140F	42	0.7818	166E
18	0.7850	60R, 88K, 159F, 279R	43	0.7817	169T
19	0.7849	61K	44	0.7814	184P
20	0.7848	144S	45	0.7811	84N
21	0.7847	38C	46	0.7807	39P
22	0.7846	189Q	47	0.7806	65N
23	0.7846	24T	48	0.7805	188R
24	0.7845	190T, 192Q	49	0.7804	28N
25	0.7840	26T	50	0.7803	218W
			51	0.7802	Baseline

Table S26 Summary of hardware configurations and runtimes for each computational protocol.

Methods	CPU model	GPU model	RAM	Run Time per Ligand
AlphaFold3 (2024 release)	AMD EPYC 7763 (128 cores, 256 threads total, up to 3.5 GHz)	NVIDIA RTX 6000 Ada (48 GB)	1.0 TB RAM	~45min (with 100 random seeds)
Boltz-2 (2024 release)	Intel Xeon Gold 6338 (64 cores, 128 threads total, 2.0–3.2 GHz)	NVIDIA GeForce RTX 3080 (10 GB)	251 GB RAM	~3min (with 10 sample per ligand)
FlexS (v5.3.1)	Intel Core i7-12700K (12th Gen, 20 threads, 3.6–5.0 GHz)	/	30 GB RAM	~8sec (with 10 samples per ligand)
Glide (Maestro v11.2)	Intel Core i7-12700K (12th Gen, 20 threads, 3.6–5.0 GHz)	/	30 GB RAM	~12sec (with 10 samples per ligand)
DiffDock (2023 release)	Intel Xeon Gold 6338 (64 cores, 128 threads total, 2.0–3.2 GHz)	NVIDIA GeForce RTX 3080 (10 GB)	251 GB RAM	~30sec (with 10 samples per ligand)

AutoDock Vina (v1.1.2)	Intel Core i7-12700K (12th Gen, 20 threads, 3.6–5.0 GHz)	/	30 GB RAM	~10sec (with 10 samples per ligand)
Gnina (2023 release)	Intel Xeon Gold 6338 (64 cores, 128 threads total, 2.0–3.2 GHz)	NVIDIA GeForce RTX 3080 (10 GB)	251 GB RAM	~7sec (with 10 samples per ligand)

Table S27 Statistical evaluation of MAE differences between AlphaFold3 and other methods for LRIP-SF potency prediction on MERS-CoV Mpro, including 95% confidence intervals, Shapiro–Wilk normality p-values, and paired t-test significance values.

	CI_lower	CI_upper	Shapiro–Wilk p value	Paired t-test p value
AlphaFold3-Glide	-0.173	-0.084	0.240	0
AlphaFold3-FlexS	-0.093	0.083	0.129	4.24E-05
AlphaFold3-Gnina_Scoring	-1.786	-1.378	0.518	0
AlphaFold3-DiffDock	-0.155	0.019	0.101	6.9E-266
AlphaFold3-Boltz-2	-0.143	0.042	0.009	3.1E-182

AlphaFold3-Boltz-2_Internal	-0.979	-0.755	0.587	0
AlphaFold3-Gnina_RR	-0.197	-0.015	0.920	0
AlphaFold3-AutoDock Vina	-0.117	-0.029	0.477	0

Table S28 Statistical evaluation of RMSE differences between AlphaFold3 and other methods for LRIP-SF potency prediction on MERS-CoV Mpro, including 95% confidence intervals, Shapiro–Wilk normality p-values, and paired t-test significance values.

	CI_lower	CI_upper	Shapiro–Wilk p value	Paired t-test p value
AlphaFold3-Glide	-0.200	-0.093	0.024	0
AlphaFold3-FlexS	-0.194	0.157	0.186	6.09E-08
AlphaFold3-Gnina_Scoring	-2.289	-1.762	0.088	0
AlphaFold3-DiffDock	-0.248	0.078	0.250	2.8E-161
AlphaFold3-Boltz-2	-0.259	0.093	0.013	7E-141
AlphaFold3-Boltz-2_Internal	-1.021	-0.705	0.516	0

AlphaFold3-Gnina_RR	-0.268	0.033	0.333	3.3E-257
AlphaFold3-AutoDock Vina	-0.120	0.000	0.617	0

Table S29 Statistical evaluation of MAE differences between AlphaFold3 and other methods for LRIP-SF potency prediction on SARS-CoV-2 Mpro, including 95% confidence intervals, Shapiro–Wilk normality p-values, and paired t-test significance values.

	CI_lower	CI_upper	Shapiro–Wilk p value	Paired t-test p value
AlphaFold3-Glide	-0.195	-0.075	0.450	0
AlphaFold3-FlexS	-0.090	0.093	0.451	0.377579
AlphaFold3-Gnina_Scoring	-4.192	-2.852	6.24E-05	0
AlphaFold3-DiffDock	-0.354	-0.147	0.121	0
AlphaFold3-Boltz-2	-0.092	0.094	0.001	4.49E-08
AlphaFold3-Boltz-2_Internal	-0.322	-0.132	0.227	0
AlphaFold3-Gnina_RR	-0.394	-0.171	0.146	0

AlphaFold3-AutoDock Vina	-0.195	-0.082	0.902	0

Table S30 Statistical evaluation of RMSE differences between AlphaFold3 and other methods for LRIP-SF potency prediction on SARS-CoV-2 Mpro, including 95% confidence intervals, Shapiro–Wilk normality p-values, and paired t-test significance values.

	CI_lower	CI_upper	Shapiro–Wilk p value	Paired t-test p value
AlphaFold3-Glide	-0.249	-0.101	0.555	0
AlphaFold3-FlexS	-0.142	0.082	0.403	2.88E-57
AlphaFold3-Gnina_Scoring	-7.188	-4.884	0.049	0
AlphaFold3-DiffDock	-0.409	-0.180	0.142	0
AlphaFold3-Boltz-2	-0.131	0.086	0.004	1.02E-17
AlphaFold3-Boltz-2_Internal	-0.359	-0.121	0.859	0
AlphaFold3-Gnina_RR	-0.491	-0.241	0.039	0
AlphaFold3-AutoDock Vina	-0.239	-0.109	0.973	0

Figure S1 Cross-docking analysis between MERS-CoV Mpro and SARS-CoV-2 Mpro. Scatter plot comparing the predicted difference of pIC_{50} (Δ_Pred) and the experimental difference of pIC_{50} (Δ_Exp) for ligands successfully modeled by AlphaFold3 against both targets. The red line represents the linear regression ($y = 0.66x – 0.39$, $R^2 = 0.29$). The RMSE between Δ_Pred and Δ_Exp is 0.64, with 84% of ligands showing matching sign of affinity change ($p < 10^{-28}$, sign test).

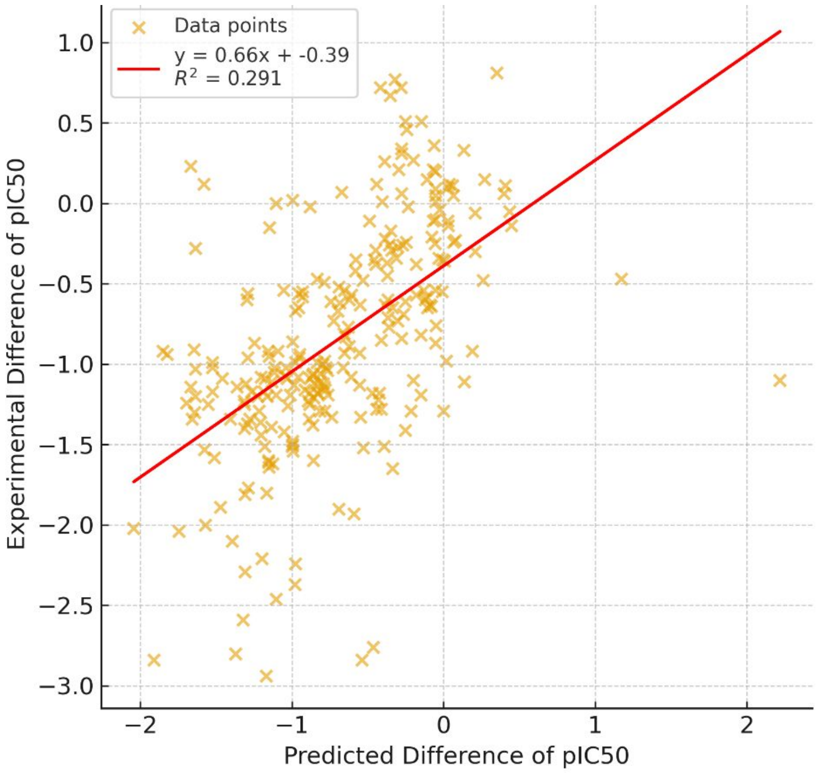

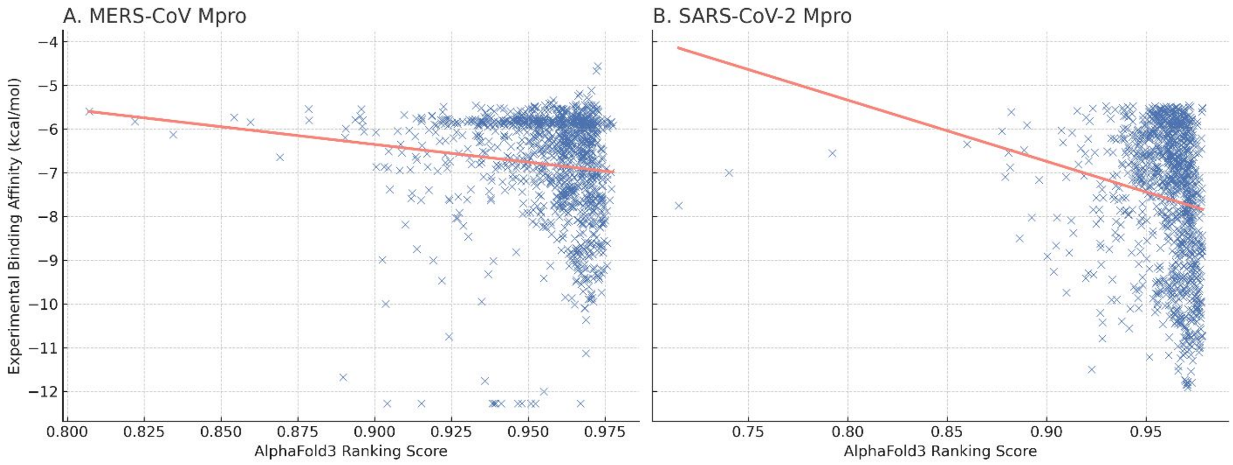

Figure S2. Correlation between AlphaFold3 ranking score and experimentally determined binding affinity. **(A)** Results for MERS-CoV Mpro. **(B)** Results for SARS-CoV-2 Mpro. Each point represents a test-set ligand, and the red line indicates the linear regression fit.
